# Comparative mRNA profile analysis from NAc of adolescent male mice after binge-like alcohol exposure eliciting deficits in context fear extinction learning

**DOI:** 10.1371/journal.pone.0322576

**Published:** 2025-06-25

**Authors:** Mario E. Lloret Torres, Arelys Rivas Jiménez, Eduardo L. Tosado Rodríguez, Kiara Cardona Jordan, Xiany X. Lay Rivera, Yaren L. Peña Señeriz, Joseph L. Capella Muñiz, Marieliz Dieppa Rodríguez, Daniel F. Ruiz Bolívar, Jovangelis González Del Toro, John A. Florian Alsina, Paola A. Colón Rivera, Jose O Garcia Colon, Abiel Roche-Lima, Cristina Velázquez-Marrero

**Affiliations:** 1 Institute of Neurobiology, UPR-Medical Sciences Campus, 201 Blvd del Valle, San Juan, Puerto Rico; 2 Integrated Informatics Services Facility RCMI-RIC-IIS University of Puerto Rico Medical Science Campus, San Juan, Puerto Rico; 3 Department Biology Cayey, University of Puerto Rico at Cayey, Cayey, Puerto Rico; 4 Department of Natural Sciences, University of Puerto Rico at Carolina, Carolina, Puerto Rico; Nathan S Kline Institute for Psychiatric Research, UNITED STATES OF AMERICA

## Abstract

**Introduction:**

Stressor-related disorders frequently co-occur with alcohol use disorder (AUD). This necessitates an understanding of the physiological and genetic factors contributing to this relationship. Binge drinking is the most common method of alcohol consumption among adolescent males and significantly increases the risk of developing comorbid stressor-related disorders and AUD. In experiments modeling the effects of a single binge-like alcohol exposure in male adolescent mice, we observed a clear deficit in context extinction learning. This exposure led to a significant initial increase in subsequent voluntary drinking on day one, as measured by the every-other-day (EOD) two-bottle choice drinking assay, which normalized thereafter.

**Methods:**

For this study we performed an mRNASeq analysis of mice nucleus accumbens (NAc), a region intricately involved in regulating both aversive contextual fear responses and reward, after EOD to profile the differential expression of mRNAs within this region. We also used immunohistochemistry of coronal brain slices to characterize expression of proteins associated with stress-related disorders and molecular alcohol tolerance, such as FKBP5, GSK3ß, and ß-catenin, within the striatum, nucleus accumbens (NAc), hippocampus, and basolateral amygdala (BLA).

**Results:**

Comparative mRNA profile analysis reveals significant long-term changes in gene expression induced by binge-like alcohol exposure, even 30 days after the initial exposure. Immunohistochemistry showed a full recovery of previously observed altered levels of target proteins prior to EOD.

**Conclusions:**

These findings suggest that the temporal activation of specific gene subsets plays a crucial role in the comorbidity of AUD and stressor-related diseases. Understanding these mechanisms can help develop more effective, integrated treatment approaches to improve outcomes for affected individuals.

## Introduction

Alcohol is one of the most widely consumed drugs among adolescents in the United States. Binge drinking is the most common method of consuming alcohol among them [1]. The Center for Disease Control (CDC) defines binge alcohol drinking as the practice of consuming 5 or more drinks on an occasion for men or 4 or more drinks on an occasion for women. The National Institute on Alcohol Abuse and Alcoholism [2] defines it as a pattern of drinking alcohol that brings blood alcohol concentration to 0.08 percent. A binge on alcohol can occur over hours, last up to several days, or in the event of extended use, even weeks, and is linked to stress-related disorder [3–5]. Binge drinking during early adolescence is related to a higher risk for AUD and more mood and stress symptoms including stress-related disorders later in life [6,7]. However, not much is understood about the factors that increase the risk of developing these conditions and even less about the neuronal, molecular, and genetic mechanisms underlying these comorbidities.

The nucleus accumbens is a functionally central structure between the amygdala, basal ganglia, mesolimbic dopaminergic regions, mediodorsal thalamus, and prefrontal cortex, it appears to play a modulative role in the flow of the information from the amygdaloid complex to these regions [8–10]. This makes it an important circuit involved in the intersection between AUD and stress-related disorders. It is further linked to psychiatric disorders such as, depression, obsessive-compulsive disorder, attention-deficit/ hyperactivity disorder, schizophrenia, and stress-related disorders, along with its well-known role in addiction [11–19]. Seminal studies also show involement of Wnt signaling in VS/NAc using models of depressive behavior [19,20], and aversive/rewarding behavior [19,21–26], demonstrating a shared region specific dysregulation of β-catenin expression across the reward pathway, especially the VS/NAc were it appears to facilitate resilience to acute stresors [27].

Our previous study on the effect of binge-like alcohol exposure prior to extinction highlighted risks for specific patterns of alcohol consumption linked to the development of stress-related disorders in adolescents [22]. These changes in behavior coincided with a decrease in FKBP5, GSK-3β, and β-catenin in the hippocampus, amygadala, and VS/NAc of the ethanol exposed mice [22]. This is consistent with our current understanding of the effects of binge-like drinking which states that at the molecular level, the binge-like exposure elicits molecular alcohol tolerance within the striatum is associated to the activation of the Wnt/β-catenin canonical signaling pathway causing persistent internalization of alcohol-sensitive large-conductance voltage- and calcium-activated channel (BK) isoform [20,22,28].

**Fig 1:**
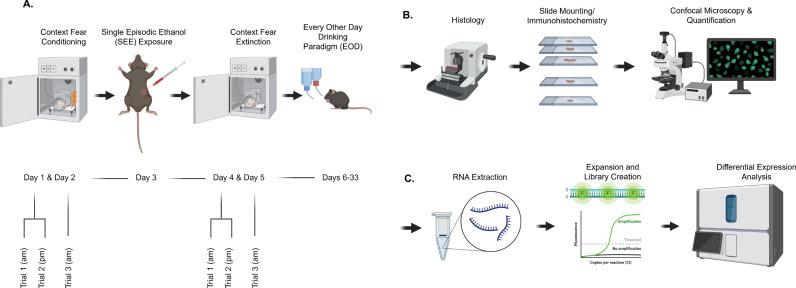
Schematic representation of methodology. (A) Behavioral fear conditioning paradigm with flowchart detailing the timing of the behavioral trials. (B) Simplified immunohistology protocol. (C) Simplified mRNA-seek protocol.

Our current study examines the protein expression of these Wnt-signaling regulators (FKBP5, GSK3β, and β-catenin) within the nucleus accumbens (NAc), hippocampus, and basolateral amygdala after EOD (Fig 1). Therefore, we report expression profiles, 28 days after extinction training, thus, representing long-term changes in response to SEE exposure. The mRNASeq analysis also indicates widespread changes in transcriptome profile within the NAc. This includes dysregulation of mRNA previously associated with trauma and stressor-related disorders [29–33] and persistent changes due to drug and alcohol exposure [34–41]. The study aims to identify convergences in gene expression profiles, potentially revealing key comorbidity pathways that sustain cellular and circuit adaptations, thereby laying the foundation for disease development. Understanding these changes can help efficiently target preventive measures, identify crucial behavioral risk factors, and design urgently needed treatments for trauma and stressor-related disorders, particularly when associated with alcohol consumption.

## Materials and methods

### Subjects

C57BL/6J adolescent (P30-P45) male mice (Jackson Laboratories, MO) were used, 120–140 mice were individually housed in ventilated cages fitted with steel lids, filter tops, and with unrestricted access to food and water on a 12/12-hour light/dark cycle. At all times, animals were treated in accordance with the National Institutes of Health, Guide for the Care and Use of Laboratory Animals [39,42]. Mice were handled and housed in accordance with the standards established by the Institutional Animal Care and Use Committee of the University of Puerto Rico Medical Sciences Campus and the National Institutes of Health. All experimental procedures were approved by the Institutional Animal Care and Use Committee (IACUC).

### Contextual fear conditioning

Fear conditioning trials were performed in a training cage [17 cm (w) x 17 cm (d) x 25 cm (h)] equipped with stainless-steel shocking grids connected to a precision feedback current-regulated shocker. Contextual fear conditioning and extinction were performed during the animal’s light cycle in a training cage within a sound attenuating chamber minimizing travel of ultrasonic vocalizations and fear-related pheromones (Stoelting Co, IL). Home cage conditions were carefully chosen and carried out consistently to minimize the negative impact on context fear conditioning learning as in [43]. Behavior, tracking, and freezing of the animal were recorded by low-light video cameras and ANY-maze software (Stoelting Co, IL). Stimulus (shock) presentation was automated by ANY-maze software. The cages were cleaned with 70% isopropyl alcohol before and after each experiment. Also, mice were tested in the same chamber using the same context in every trial. Before the mice passed through any behavior test, they had a period of 45–60 minutes for acclimation. Protocol followed closely that described in [44]. Briefly, mice were placed in the above-mentioned chamber and received one 2-second, 0.7mA foot shock 136 seconds into the trial. The trial total time was 228 seconds. After each trial they were returned to their home cages with water and food ad libitum (Fig 1A).

### Single Episode Ethanol (SEE) exposure

Prior to SEE mice were equally distributed by weight and freezing behavior learning during the Contextual Fear Conditioning phase, between the saline N = 26 (control group) and ethanol group N = 28. One of the main limitations in the study of binge drinking is that animal models will not voluntarily drink the necessary amount to reach the required blood alcohol concentrations (0.08%) [1]. To ensure mice achieve the appropriately high concentration of blood ethanol concentrations mice received six intraperitoneal injections (i.p.) of 20% EtOH v/v with saline solution using a 26.5-gauge needle evenly spaced over 6 hours, after contextual fear conditioning trials (Fig 1A), as previously described [25]. The first injection was at an EtOH concentration of 1.8 g/kg, and the subsequent five injections were at a concentration of 1.2 g/kg. After each injection, mice were placed back in their housing and carefully monitored. Blood alcohol concentrations are known to return to naïve levels after 3hr withdrawal [25].

### Contextual fear extinction

Previous studies demonstrated that differences in context fear extinction can be observed after 3 trials, with a single shock in each trial [22,44]. Context fear extinction trials were performed in a designated chamber with Context A, without the administration of the shock (the same context as the conditioning). During extinction trials, mice remained in the chambers for 3.8 minutes (228 seconds) and then returned to their home cages. They received three extinction trials in total (Fig 1A).

### Two-bottle choice, every-other-day drinking

For the EOD protocol, 30 animals (Ethanol: N = 16, Saline: N = 14) were selected from the previous cohort to continue the experiment. Two bottles were prepared for each mouse: Bottle 1 contained a 20% EtOH v/v H₂O solution, and Bottle 2 contained H₂O. Each solution was transferred into 50-mL polystyrene centrifuge tubes, sealed with rubber stoppers, and fitted with straight stainless steel sipper tubes. The tubes were placed in the steel lids of the cages, equipped with filter tops, and positioned equidistantly. The solutions were replenished every four days on designated Choice Days during which the positions of the bottles were alternated to prevent side bias. Between Choice Days, the experimental bottles were removed, and each cage was provided with a single standard drinking water bottle supplied by the Animal Resource Center of the University of Puerto Rico Medical Sciences Campus.

The complete EOD protocol lasted 27 days, consisting of 14 Choice Days and 13 No-Choice Days. The first day following the extinction trials was designated as Choice Day 1. Body weights of the mice were recorded every 72 hours, while ethanol and water intake values were measured every 24 hours to the nearest 0.2 mL. These data were used to calculate self-administered ethanol doses (g/kg), water consumption (mL), and the relative preference for ethanol (20% ethanol intake/total fluid intake) (Fig 1A, 2A). Mice were euthanized 24 hours after completing the EOD protocol.

**Fig 2:**
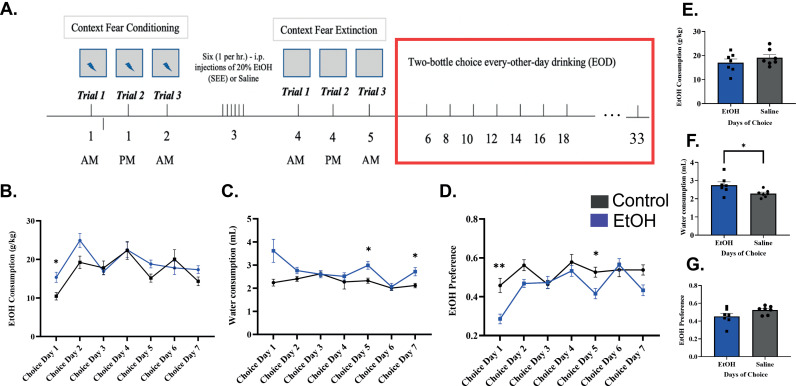
Two bottle choice every other day drinking task – EOD. (A) Diagram displaying simplified EOD paradigm. (B-D) Line graphs detailing drinking pattern during first seven EOD Choice Days measuring; EtOH consumption (B), Water consumption (C), and EtOH preference (D). (E-G) Bar graphs quantifying the total average EtOH consumption (g/kg), Water consumption (mL) and EtOH preference (20% ethanol intake/total fluid intake) for all 14 Choice days. Statistical significance was determined using One-way ANOVA followed by a Bonferroni test * p ≤ 0.05 in the line graphs. Students t-test was used to determine changes in the average consumption (Bar Graphs) * p ≤ 0.05. Data presented as mean and SEM.

### Paraffin embedding

After euthanasia, a total of 6 brains (N = 3 Ethanol, N = 3 Saline – Three brain slices (replicates) per brain of each region were assayed for a total of 9 slices per treatment) brains were fixed using Paraformaldehyde 4% solution in PBS (CAS 30525-89-4) using cardiac perfusion under xylazine/ketamine cocktail anesthesia. After fixation, brain tissue was dehydrated using 50, 75, 95, 100% ethanol solution (Sigma Aldrich CAS 64-17-5) and xylene (Sigma Aldrich 1082984007), immersed in paraffin (Thermo Scientific 22-900-701) at 60°C for 12 hours (paraffin was changed every 4 hours) using the protocol stated in Zhanmu O et al., 2019 [45]. After dehydration and paraffin immersion, brain tissue was embedded in paraffin blocks using Epredia™ HistoStar™ Embedding Workstation (Cat. No. A81000002) and were left at 4^°^C until used (Fig 1B).

### Immunohistochemistry

After paraffin embedding brains were sliced using a fully motorized rotary microtome (Leica, RM2250), and slices of the dorsal hippocampus (dHPC), basolateral amygdala (BLA), and nucleus accumbens core (VS/NAc) brain tissue were collected (5-μm-thick). After all brain slices were made, they were deparaffinized and rehydrated as seen in Zaqout et al., 2020 [46]. For permeabilization slides were given 2 washes with PBS 1X (2 min), then one wash with PBS 1X (10 min), and finally 2 washes with PBS/Gelatin/Triton 0.25% (10 min). Slides then underwent blocking with a 5% BSA solution for 1 hour. After blocking were incubated with primary antibodies for FKBP5 (1:500) (Invitrogen 711292), ß-catenin (1:200) (ab32572), GSK-3ß (1:500) (ab93926) and NeuN (1:500) (ab104224 & ab177487) overnight. The next day slices were washed twice with PBS 1X (10 min), then one wash with PBS/gelatin/triton 0.25% (10 min). They were then incubated with secondary antibodies, Goat Anti-Mouse Alexa Fluor 488 (ab150113), Goat Anti-Rabbit Alexa Fluor 488 (150077), mounting medium with DAPI (ab104139), Goat Anti-Mouse Alexa Fluor 594 (ab150116) and Goat Anti-Rabbit Alexa Fluor 594 (ab150080). The dilution used for all secondary antibodies was 1:500.

### Confocal microscopy acquisition and analysis

FKBP5, ß-catenin, and GSK-3ß expressions were measured by a tissue’s fluorescence intensity and were viewed using Nikon Instruments A1 Confocal Laser Microscope using a 10X magnification and analyzed with Nikon Nis Element Advance Research program using the maximum intensity projection (MIP) on all images (Fig 1B).

### Brain region isolation and RNA extraction

For bioinformatics analysis, a cohort of animals was selected, and following the completion of behavioral testing, these mice were euthanized by rapid cervical dislocation. The brains were removed, placed in a cold matrix, and coronally sliced. 2 mm brain slices containing the ventral and dorsal striatum were isolated. Subsequently, a 0.84 mm diameter stainless steel micropunch was used to remove bilateral samples from the NAc core of each C57BL/6 mouse (18 mice total) which were placed into 1.5-ml tubes. Tissue punch samples were immediately snap-frozen, and then stored at −80°C. RNA extraction was done by placing samples into ice-cold TRI reagent, 1 ml per 50–100 mg of tissue of TRIzol reagent (Sigma-Aldrich). Total RNA was extracted following the manufacturer’s instructions (mRNeasy Micro Kit; QIAGEN). RNA was quantified by UV spectrometry with a NanoDrop^TM^ UV-Vis Spectrophotometer (Thermo Scientific, United States). Total RNA was shipped to Psomagen Laboratories where mRNA was further isolated and processed with the highest standards to determine the quality of samples for subsequent RNA sequencing.

### RNA sequencing

For library construction, mRNA was extracted from each sample and underwent quality control (QC). Qualified samples proceeded to library construction. The sequencing library was prepared by random fragmentation of the DNA or cDNA sample, followed by 5’ and 3’ adapter ligation. Alternatively, “tagmentation” combines the fragmentation and ligation reactions into a single step increasing the efficiency of the library preparation process. Adapter-ligated fragments are then PCR amplified and gel purified. For cluster generation, the library was loaded into a flow cell where fragments were captured on a lawn of surface-bound oligos complementary to the library adapters. Each fragment is then amplified into distinct, clonal clusters through bridge amplification. When cluster generation is complete, the templates are processed for sequencing. We utilized Illumina SBS technology allowing for a reversible terminator-based method that detects single bases as they are incorporated into DNA template strands. As all 4 reversible, terminator-bound dNTPs are present during each sequencing cycle, natural competition minimizes incorporation bias and greatly reduces raw error rates. The result was highly accurate base-by-base sequencing that virtually eliminates sequence-context-specific errors, even within repetitive sequence regions and homopolymers.

### Raw data

Sequencing data is converted into raw data for the analysis. The Illumina sequencer generates raw images utilizing sequencing control software for system control and base calling through an integrated primary analysis software called RTA (Real Time Analysis). The BCL (base calls) binary is converted into FASTQ utilizing the illumina package bcl2fastq. Adapters are not trimmed away from the reads. The total number of bases, reads, GC (%), Q20 (%), and Q30 (%) are calculated for the 6 samples. For example, in NAcCore-Control-1, 98,998,428 reads are produced, and total read bases are 14.9G bp. The GC content (%) is 51.23% and Q30 is 93.41%. Note: N = 3 NAc core control and N = 3 experimental. Each N contains the NAc core tissue punches from 3 mice for a total of 9 tissue punches per N.

### Phred quality score

Samples were processed for quality control using a Phred quality score that numerically expresses the accuracy of each nucleotide. A higher Q number signifies higher accuracy. For example, if Phred assigns a quality score of 30 to a base, the chances of having base call errors are 1 in 1000. Phred Quality Score Q is calculated with −10log10P, where P is the probability of an erroneous base. These tests were performed using an independent agency and according to strict guidelines (Psomagen, MD).

### Bioinformatics—Differential gene expressions analysis

The paired sequence reads for control and EtOH samples were inspected for quality and trimmed using the Trim Reads tool from the CLC Genomics Workbench® software version 22.0.5, using a quality trimming score of 0.05. The bioinformatics analysis was performed to identify Differential Expressed Genes (DEGs) across groups using the bioinformatics software, CLC Genomics Workbench®. The reference genome was the Mouse (*Mus musculus*) reference genome GRCm39.104 acquired from CLC Reference Sets and Ensembl data repository, including genome annotations. The statistical analysis was performed using the option “*Differential Expression in Two Groups”* from the CLC Genomics Workbench® with the “*TMM normalization*” method, as a parameter. The final DEGs were considered as these genes with a Fold change (FC) ≥ |2| and p-value ≤ 0.05.

### Gene functional, pathway and network analyses

The list of DEGs from the statistical analysis was used as input for the gene enrichment and interaction network analysis for the Molecular Signatures Database (MSigDB v7.1) and Ingenuity Pathway Analysis Software [47]. The MSigDB was used to identify Gene Ontologies (GOs) and pathways using as an input the up and down-regulated genes altered by candesartan and vehicle after TBI at 3 dpi in the hippocampus. GO function analysis and pathways were identified using a Fisher’s exact test with a false discovery rate of less than 0.05. Secondly, the DEGs were annotated using the Ensemble Accession identification during the IPA enrichment analysis. An Ingenuity CORE analysis was performed for the enrichment, and identification of the canonical pathways, and diseases/functions associated with the DEGs. As a result, we considered those canonical pathways and diseases/functions with p-values ≤ 0.05. In addition, interaction networks associated with alcohol, drug dependencies, and trauma and stressor-related disorder were performed using the IPA software and the DGEs as input.

The Gene-Gene interaction networks were generated by selecting only the differentially expressed genes of this analysis. The criteria to observe Gene-Gene interactions was to only identify annotated protein interactions between the differentially expressed genes, the genes with the highest number of interactions were at the center of the network. The Pathway-Gene interaction network was generated by selecting enriched pathways of importance for our hypothesis associated with drug and alcohol dependence. A total of 7 pathways were considered of highest importance based on enrichment p-value, and number of genes associated per each pathway. The causal network between trauma and stressor-related disorder, stress-related disorder, and drug and alcohol dependence were generated by using the Ingenuity Pathway Knowledgebase search engine. The keywords used to search for matching terms were “Drug Dependence”, “Alcohol dependence”, “Anxiety” and stress-related disorders, under ontological categories of “Organismal Injury and Abnormalities”, “Psychological disorders”, and “Neurological Diseases”, under Ingenuity Knowledgebase Content Version: 73620684 (Release Date: 2022-03-12).

### Statistics

Statistical analysis for behavior and immunohistochemistry was generated using GraphPad Prism 7.01 and using repeated measures two-way ANOVA or mixed model followed by a Bonferroni multiple comparison test. The confidence level was set to 0.05 (*p-value*), and all results are presented as the mean ± standard error of the mean [47]. Significances of these tests are displayed as indicated: * p ≤ 0.05, **p ≤ 0.01, *** p ≤ 0.001 and **** p ≤ 0.0001.

### Ethics statement

The methods used in this study were approved in writing by the University of Puerto Rico’s Institutional Animal Care and Use Committee (IACUC) IACUC as Protocol A830117- Effects of Alcohol Molecular Tolerance on Fear Extinction.

## Results

### Two bottle choice every other day drinking

Mice underwent EOD to determine the effects of binge-like alcohol exposure on voluntary drinking. No significant differences were observed in average ethanol drinking between binge drinking and control mice over the 7 drinking days, two weeks total (Fig 2E). However, there was a significant increase in overall drinking on the first trial day, mixed model (REML) and Bonferroni’s test showed higher drinking in binge drinking animals as opposed to controls p < 0.05 (Fig 2B) suggesting binge-like alcohol exposure may induce early increased ethanol intake. Interestingly, water consumption was also found to be significantly increased in the ethanol group on average (Fig 2F), Student’s t-test showed p < 0.05. This result was caused primarily by a significant increase in water consumption on trial days 5 and 7 for ethanol exposed mice, mixed model (REML) and Bonferroni’s test showed p < 0.05 (Fig 2C). This increase in water consumption may be the result of increased withdrawal symptoms [48]. When evaluating ethanol preference, no significant changes were observed on average (Fig 2G) between control and ethanol exposed. Increased ethanol preference was only observed within the control group on trial days 1 and 5, mixed model (REML), and Bonferroni’s test showed p < 0.05 (Fig 2D). The differences on day one are likely caused by the novelty effect as control animals had not previously been exposed to ethanol.

### Immunohistochemistry (IHC)

**Fig 3:**
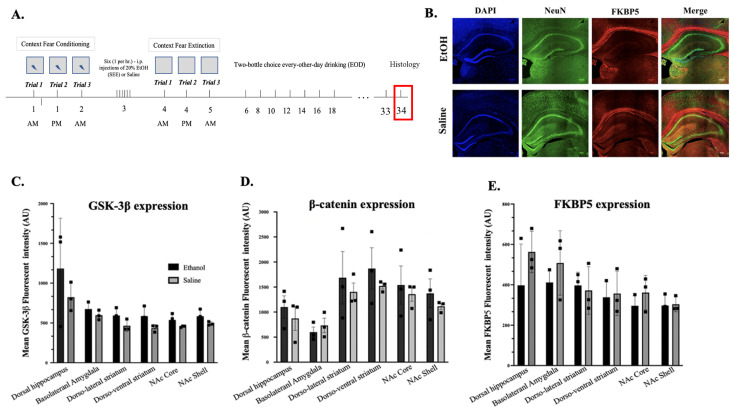
Relative expression of EtOH-impacted proteins in key reward circuit areas. (A) Diagram detailing simplified paradigm for immunohistochemistry. (B) Examples of immunohistochemical images (hippocampus) for ethanol and control groups. (C-E) Bar graphs showing the relative |expression of alcohol-associated genes (GSK-3β, β-catenin and, FKBP5) in various reward circuit-associated regions including dorsal hippocampus, basolateral amygdala, dorso-lateral striatum, dorso-ventral straitum, NAc Core and NAc Shell.

Upon completion of the behavioral paradigms, IHC was performed on a subset of mice to assess changes in β-catenin and regulatory proteins GSK-3β and FKBP5 [49,50] (Fig 3A). This was done to detect changes in β-catenin-induced development of molecular alcohol tolerance in several reward system-related regions such as the striatum, basolateral amygdala, and hippocampus [20,21] (Fig 3B). Mean intensity of β-catenin, FKBP5, and GSK-3β showed no significant differences in the dorsal hippocampus, basolateral amygdala, dorso-lateral striatum, dorso-ventral striatum, NAc core, or NAc shell (Fig 3 C–E). This suggests that while binge-like alcohol exposure can affect context extinction learning behavior, specific changes in protein expression may be limited to early time windows and have the potential to fully recover over time.

### Differential gene expression analysis

**Fig 4:**
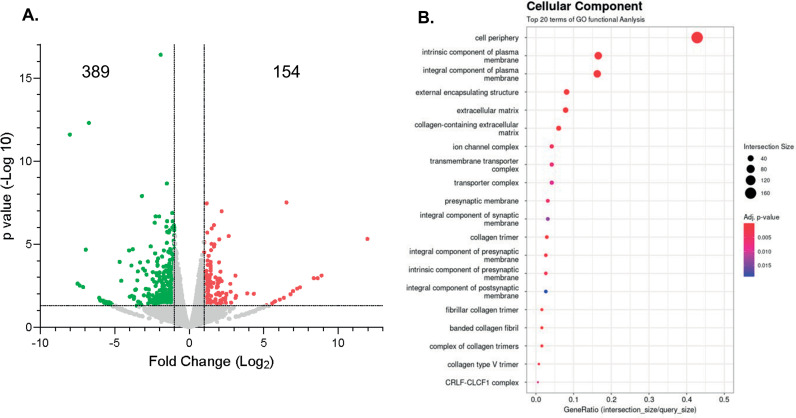
Differential gene expression and cellular components associated with differentially expressed genes. (A) Volcano plot displaying the p-value of the differentially expressed genes, statistical cutoff established as (Log2 FC [1]; -log10 p value = 1.3). (B) Graph representing the top 20 significantly impacted Cellular Components containing differentially expressed genes, sorted by number of genes, and statistical significance with a p-value of p < 0.05.

A total of 524 genes were determined to be statistically differentially expressed out of a total of 18,092 genes examined. Of these, 153 genes were up-regulated, and 371 were down-regulated (Fig 4A). The top 40 most significant genes included two potassium channel proteins, the upregulation of KCNG3 gene (potassium voltage-gated channel modifier subfamily G member 3) whose functions include, neurotransmitter release, heart rate regulation, insulin secretion, neuronal excitability, epithelial electrolyte transport, smooth muscle contraction, and cell volume control; and the downregulation of KCNJ13, a member of the inwardly rectifying potassium channel family. Other genes notably associated with epigenetic regulation include the upregulation of the H4 clustered histone 13 [51] and downregulation of H2A clustered histone 11, both replication-dependent histones. G-protein associated proteins are strongly represented such as the GPR39 gene (G protein-coupled receptor 39) involved in, zinc-dependent signaling in epithelial tissue in intestines, prostate, salivary gland secretion and pathophysiology of depression [52].

### Gene functional, pathway and network analyses

As a result of the GO function analysis, three separate groups were created. The first group was associated with “Cellular Components” where the top 20 terms of GO included genes from our DEGs are shown in Fig 4B. Some of the most relevant terms included “cell periphery”, “intrinsic component of plasma membrane” and “integral component of plasma membrane” as well as terms associated with molecular alcohol tolerance such as “ion channel complex” and “transmembrane transporter complex”. The second group was related to “Biological Process” where the top 20 terms of GO functional analysis included “ion transport” with more than 50 DEGs from our list and p-value < 0.01, “transmembrane transport”, “cation transport”, “ion transmembrane transport”, as can be seen in Fig 4B.

**Fig 5:**
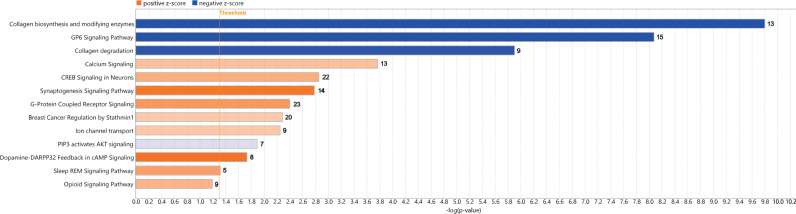
Pathways significantly impacted by alcohol exposure. Graph representing upregulated or downregulated (blue) pathways ordered by p-value and displaying the number of differentially expressed genes (right-hand side of each column).

The gene ontology associated with biological process (Fig 5) showed the single highest significance is ion binding closely followed by specifically, cation binding and metal ion binding [53–55]. These correlate well with the gene ontology associated with molecular function data highlighting binding of ions and metals as the most significant (Fig 3C). Known correlates for cation binding within the nucleus accumbens (NAc) include dopamine binding [56–61] and drugs of abuse such as cocaine [61]. The NAc is 95% composed of medium spiny neurons (MSNs), that are typically divided into those that express dopamine receptor D1 (D1R, D1-MSNs), and those that express dopamine receptor D2 (D2R, D2-MSNs) [62] with evidence supporting the expression of the D3 [63], D4 and D5 receptors [64,65]. The regulation of downstream events after specific dopamine receptor activation within the NAc is critical in determining reward versus aversive responses [66–68]. Furthermore, binding of magnesium ions is an important regulatory element in alcohol molecular tolerance of BK channels [69–73]. For a review of other ions and metals affected by alcohol exposure see [74].

Pathways like phagosome formation, protein-coupled receptor signaling, and CREB signaling in neurons have been associated with ethanol consumption within the Nucleus accumbens (NAc) (Fig 5) [75,76]. Ethanol exposure and consumption have been shown to lead to apoptosis and autophagy in macrophages of rats. Ethanol has been further related to the suppression of the phagosomal adhesion maturation process, leading to bacterial phagocytosis [75]. There is also clear evidence of changes associated with G-protein expression in response to alcohol exposure. Chronic alcohol drinking has been related to the hypermethylation and downregulation of the G-protein coupled receptor 39 (GPR39) gene [77]. The role of this gene is crucial in the encoding of the Zn^2+^-binding metabotropic receptor which is known for the modulation of inhibitory and excitatory processes in neurotransmitters, altering the balance of diseases like AUD [78]. Studies have investigated the potential use of a GPR39 agonist for the reduction of alcohol consumption in a preclinical rodent model. Their results showed a mechanism for the agonist in which the increase in alcohol consumption was prevented and decreased drinking in mice after acute administration of it, which could occur by the processes of excitatory neurons in the NAc. Furthermore, the activation of Protein Kinase A and its subunits in the NAc and Ventral tegmental area within the brain are due to the consumption and exposure of addicting drugs like nicotine and ethanol. Research by Pandey and colleagues showed that the NAc and the CREB signaling has a role in Ethanol drinking behaviors which could affect positively and negatively due to the influence of alcohol use and abuse [79]. Also, these results provide novel evidence that decreased CREB signaling expression in neurons may regulate alcohol-drinking behaviors via the decreased expression of NPY in the NAc shell [80].

### Gene interactions

**Fig 6:**
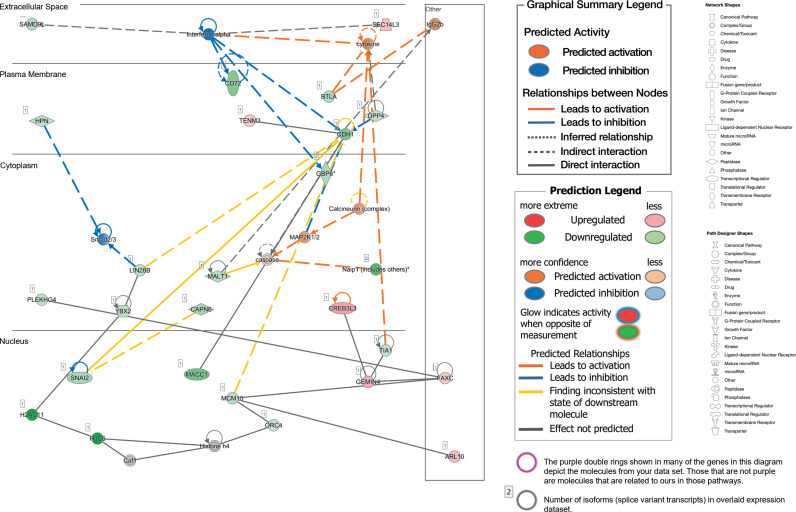
The Gene-Gene Networks. The networks were generated using Ingenuity Knowledgebase which divided gene interactions into three clusters designated networks I-III, based on direct interactions between the genes differentially regulated by SEE exposure.

The Gene-Gene Network generated using Ingenuity Knowledgebase was divided into three clusters (Fig 6), based on direct interactions between the genes. Genes interacting within network-II include the genes OPRM1, CDH1, PCDHGB1, and TRIM54, as predominant based on Ingenuity knowledge base [Refseq entry 4988, Oct 2013, and Uniprot accession P35372], Highlighted is the Mu-Type Opioid receptor (OPRM1) which is the principal target of endogenous opioid peptides and opioid analgesic agents such as beta-endorphin and enkephalins. This receptor also has an important role in dependence to other drugs of abuse, such as nicotine, cocaine, and alcohol via its modulation of the dopamine system. The gene Protocadherin gamma subfamily B1 (PCDHGB1) [Refseq entry 56104, Jul 2008, and Uniprot accession Q9Y5G3] belongs to the neural cadherin-like cell adhesion proteins, these proteins may play a critical role in the establishment and function of specific cell-cell connections in the brain. The gene Cadherin-1 (CDH1) [Refseq entry 999, Nov 2015, Uniprot accession P12830] is a member of the calcium-dependent cell adhesion proteins [81]. According to Cheon and colleagues, CDH1 is involved in mechanisms regulating cell-cell adhesions, mobility, and proliferation of epithelial cells. The Gene Tripartite motif-containing protein 54 (TRIM54) [Refseq entry 57159, Jul 2008, Uniprot accession Q9BYV2], which, in conjunction with RNF28, and RNF29 form heterodimers that may be important for the regulation of titin kinase and microtubule-dependent signal pathways in striated muscles.

**Fig 7:**
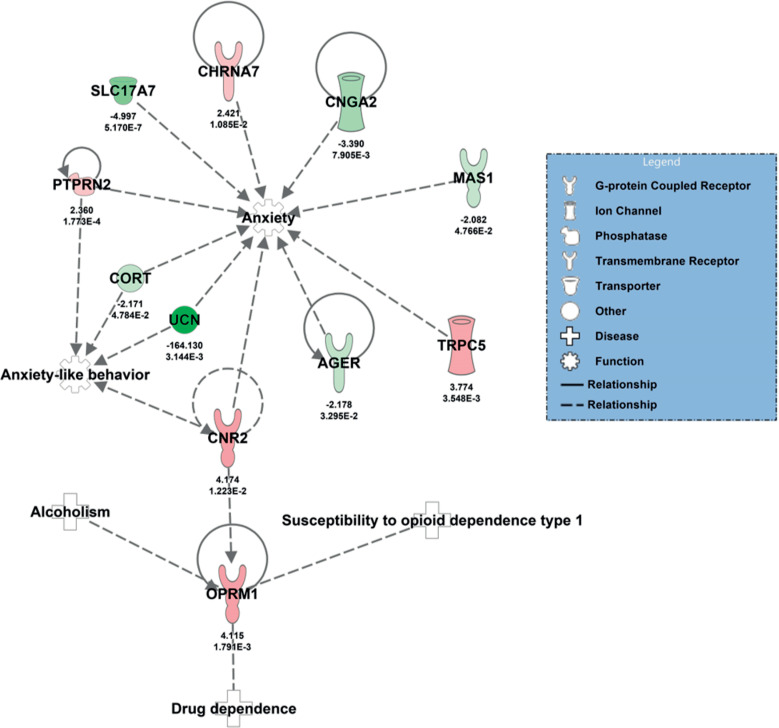
Differentially expressed genes related to anxiety and addiction. Diagram listing genes associated with anxiety and addiction displaying a link through the OPRM1 gene. A total of eight genes were identified interacting with trauma and stressor-related by causation (AGER; TRPC5; SLC17A7; UCN; PTPRN2; CHRNA7; CNGA2 and MAS1) [93], and four genes were found associated with trauma and stressor-related behavior (CNR2; UCN; PTPRN2 and CORT) [94,95].

The Pathway-Gene interaction network was generated by considering the top three most enriched pathways based on an enrichment threshold of p-value ≤ 0.05. These pathways include G protein-coupled receptor signaling, CREB signaling in neurons, and phagosome formation. Additionally, two more pathways were included in this network: the Wnt/β-catenin signaling pathway and the dopamine-DARP32 feedback in the cAMP signaling pathway (Table 1; S1–S9 Figs; S1–S9 Tables) [82]. The gene OPRM1 was identified in correlation with annotations related to drug and alcohol dependencies [83,84] and gender differences in response to binge drinking in adolescent mice [85–87]. Notably, it is linked specifically to the development of trauma and stressor-related behaviors and ethanol consumption in mice [88]. Annotations from the Ingenuity knowledge base suggest that mutations at positions 118A > G (rs1799971) may increase susceptibility to drug and alcohol dependence [89]. This finding has also been observed in recent pharmacogenetic studies involving the variant rs1799971 from OPRM1 [89–92]. The Functions-Genes interaction network between trauma and stress-related disorders (Fig 7) was similarly generated by considering terms from the Ingenuity Knowledge Base associated with trauma and stress-related disorders. This network incorporates several highlighted genes from the gene interaction analysis.

**Table 1:** Examples of differentially expressed EtOH vs. Control Wnt-associated genes.

Gene name	Protein	Function/Association with Wnt Signaling	Reference
**AKAP9**	A-Kinase anchoring protein 9	Akap9 silencing reduces Wnt/beta-catenin signaling.	[Wu et al., 2022]; [Sillibourne et al., 2002]
**CDH1**	E-Cadherin	CDH1 decreases activity of Wnt signaling, E-Cadherin expression increases with the suppression of Wnt signaling.	[Liu et al., 2018]; [De Re et al., 2022]; [Cong et al 2013].
**DKK1**	Dickkopf-1	DKK1 inhibits Wnt/β-catenin and Wnt/LRP6 signaling.	[Chen et al., 2021]; [Li et al., 2010]; [Semënov et al., 2008]; [Davidson et al., 2002].
**HCAR1**	Hydrocarboxylic Acid Receptor 1	Wnt signaling-related genes, i.e., Wnt3A, Wnt7A & Wnt10A were downregulated in GPR81/HCAR1 (-/-) cells.	[Madaan et al 2019]; [Lee et al., 2018].
**PCDH18**	PDC18	PCDH18 depletion promotes nuclear beta-catenin accumulation and translation.	[Zhou et al 2017]; [Zhao et al., 2023].
**TPBG**	Trophoblast Glycoprotein	Inhibits Wnt/beta catenin signaling and activates non canonical Wnt pathways.	(Kagermeier- Schenk et al., 2011]; [He et al., 2015]; [Zhao et al., 1993].
**TWIST1**	Twist Transcription Factor	Canonical Wnt signaling induces Twist1, TWIST1 enhances beta-catenin degradation.	[Reinhold et al., 2006]; [Liu et al., 2022]; [Yu et al., 2020].

Differentially expressed genes related to anxiety and addiction were diagramed using ingenuity pathway (Fig 7). The diagram lists genes associated with anxiety and addiction displaying a link through the OPRM1 gene. A total of eight genes were identified interacting with trauma and stressor-related by causation (AGER; TRPC5; SLC17A7; UCN; PTPRN2; CHRNA7; CNGA2 and MAS1) [93], and four genes were found associated with trauma and stressor-related behavior (CNR2; UCN; PTPRN2 and CORT) [94,95]. The results from the network suggest most of the causal interactions are by knockout studies in mice. Annotations between anxiety and the gene AGER, suggest that 129S1/SvImJ mutant of the Trpc5 gene in mice (allele Trpc5tm1.1Clph/Y, by Knockout), decreases anxiety in mouse [96]. Knockout of the gene AGER in mice suggests a decrease in anxiety in mice [97]. Knockout of the gene SLC17A7 in mice suggests an increase of anxiety in mice 129 * C57BL/6N SLC17A7 mutant mouse [98]. Knockout of the gene CORT in mice increases anxiety-like behavior [99]. Knockout of the gene PTPRN2 in mice increases anxiety-like behavior [100]. In lateral septum from male rats infusion of the UCN gene increases anxiety-like behavior [101]. Knockout of the gene CHRNA7 in129S7/SvEvBrd * C57BL/6J mouse cells, suggests a decrease in anxiety [102]. Knockout of the gene MAS1 in 129P2/OlaHsd mouse cells, suggests an increase in anxiety [103]. In male mice, blockade of CNR2 protein by AM630 increases anxiety-like behavior by male mice that is decreased by mutant mouse Fmr1 gene by knockout [104].

## Discussion

Comparative transcriptomics is essential in understanding comorbid diseases such as Alcohol Use Disorder [105] and stress-related disorders because it enables researchers to identify common molecular pathways and gene expression profiles impacted during disease development within specific brain regions. The convergent genes may contribute to the development and maintenance of each disorder and their comorbidity. Comparative studies can also identify biomarkers indicative of susceptibility to AUD, stress-related disorders, or both. These biomarkers are useful for early diagnosis, monitoring disease progression, and evaluating treatment effectiveness. Additionally, longitudinal transcriptomic studies can provide insights into how gene expression changes over time in response to stress, alcohol exposure, or treatment. These and future approaches are designed to enhance our understanding of the progression of comorbid AUD and stress-related disorders, identify critical windows for intervention, and inform the development of personalized therapeutics. Our results observed early β-catenin dependent BK surface expression redistribution after binge-like alcohol exposure, linked to fear-extinction deficits [20]. These changes are presumed to be a part of a chain reaction leading to long-term gene expression changes most likely via TCF/LEF transcriptionally regulated genes. It also provides evidence that a single session of binge drinking, prior to fear extinction, is sufficient to chart a distinct profile of long-term transcriptional changes unique from those observed with EOD alcohol exposure only.

### Voluntary drinking and molecular alcohol tolerance

During every-other-day drinking (EOD) observations, differences were notable on the first-choice day. On this day, we observed a significant increase in ethanol consumption, a non-significant trend towards increased water consumption, and a decreased ethanol preference in the binge-like alcohol group compared to saline controls. The increase in EtOH consumption on Day 1 is unlikely to be attributed to freezing levels during conditioning, as these were comparable between treatment groups, as previously reported. Furthermore, open field tests assessing anxiety during conditioning and extinction trials revealed no differences in center vs. periphery time or locomotion [22]. Additionally, extinction deficits do not reliably predict increased alcohol consumption in mice [106]. Instead, the novelty effect likely influenced changes in preference, as control mice had not been previously exposed to ethanol [106–109]. Interestingly, delays in auditory fear extinction have been linked to increased alcohol consumption but only in female rats [107]. Future experiments will allow us to elucidate this possibility. However, it is also possible that frequency and/or strength of the single foot shock per trial may not be able to elicit the levels of anxiety that could be linked to augmented voluntary consumption [108,110–112]. Interstingly, the exception was water consumption, which significantly increased in the ethanol group on days 5 and 7 in mice *SEE* treated, which may have resulted from a counteractive measure in response to increased EtOH exposure. These differences, along with the trend on day one, may have contributed to a significant increase in average water consumption linked to withdrawal symptoms [48]. This is consistent with some of the differentially expressed genes found in the bioinformatic analysis [113–115]. Linking voluntary drinking to known changes in Wnt/β-catenin activation in response to binge-like alcohol exposure, we measured protein expression via immunohistochemistry analysis. We examined differences in the expression of β-catenin and some of its regulatory proteins to determine if the mechanism of early molecular alcohol tolerance [20,21] was present. The lack of clear behavioral voluntary drinking differences despite molecular changes could stem from the complexity of the relationship between molecular alterations and behavior, the limitations of the experimental paradigm, or compensatory mechanisms in response to ethanol exposure. These molecular changes can further be the bases for future susceptibility to stress and/or alcohol-related behaviors. Further analysis or adjustments to the experimental design may help clarify these observations and highlight behavioral impacts due to *SEE*. For example, it has yet to be determined if any of these will impact alcohol consumption under repeated exposure [116] or increase the probability of dependence in adulthood [117]. Future studies will further analyze other protein targets which could mediate the effects of binge-like alcohol expousure on extinction and/or alcohol consumption through opioid signaling [118], cannabinoid signaling [119–121] and dopamine receptors such as the D1-receptor [122–124].

### Long-term alcohol and plasticity

In terms of our bioinformatic analysis, a large subset of the over 200 differentially expressed mRNA’s are known to be associated with long-term alcohol exposure [38]. This includes several GPCR’s (ADGRF4, ADGRG5, GPR39…) which are known to play roles in the development of withdrawal and relapse and whose expression impacts alcohol consumption [77,125]. Unsurprisingly GPCR signaling were significantly altered, being the second most prevalent with 23 genes many of which (APCY9, CREB313, SHC3) which are themselves associated with AUD [126–128]. One notable finding was an increase in phosphodiesterase 2A (PDE2A) whose inhibition was shown to attenuate AUD induced by stress-related disorders suggesting that binge drinking during fear-conditioning could create vulnerabilities for future chronic drinking [129]. We also found increased Hydroxycarboxylic acid receptor (HCAR) expression, a G protein-coupled receptor (GPCR) which binds to lactic acid. HCAR is known to increase β-catenin expression in a zebrafish model suggesting a connection to the molecular alcohol tolerance hypothesis which requires β-catenin signaling [130]. These results are not unexpected, as numerous studies have provided evidence of the roles of GPCRs in addictive behaviors [131]. Multiple alcohol-associated genes not related to GPCR signaling (AP1G2, CNR2, ITGA10, ITGA2, ITGAE, MYO5C, NMUR2…) are found in to be differentially expressed. These genes were entwined with alcohol during multiple processes such as development, drug seeking, and drug taking [132–138]. As animals from all groups underwent chronic drinking this suggests that binge-like alcohol exposure administered prior to extinction of fear conditioning could predispose mice to drinking by way of aberrant synaptic trafficking. This represents the long-term effect of neurochemical changes that caused changes in behavior a month prior. Interestingly the PLA2G4B gene was found to be increased as it had previously been observed during binge drinking suggesting it may serve as a marker weeks after the binge event itself [139]. Some of our other most significantly impacted pathways, CREB signaling formation with 22 genes and Breast Cancer regulation with 20 genes, had a high degree of overlap with GPCR signaling. As GPCR’s are known for directly regulating CREB through cAMP and PKA this result is not unexpected [140]. The only nonoverlapping gene was CACNA1 which is itself differently expressed in the mouse amygdala after chronic alcohol [141]. The same can be said for breast cancer regulation where GPCR’s are one of the most studied genes involved in tumorigenesis and one of the most promising targets for treatment-resistant triple negative breast cancers [142]. We found only one non-overlapping gene PPP1R3A whose involvement in PIP makes a prominent target for addictive behavior in the accumbens [143], but also a potential regulator of BK channel activity [144]. GPCRs are known to play key roles in the development of synaptic plasticity suggesting binge-like drinking could be disrupting these learning processes [145].

We also found a large group of genes associated with alcohol-linked diseases (CR2, GRPR, MYH6, SCARA5….). These genes, known to play roles in liver and heart conditions associated with AUD [146–149] were present in multiple pathways. Perhaps the most prominent example of overlapping genes was the subset of differentially regulated collagens (COL11A1, COL16A1, COL22A1, COL24A1, COL27A1, COL2A1, COL5A1, COL5A3, COL6A1, COL6A6, COL7A1, COL9A1) as these were present in multiple pathways such as the Collagen biosynthesis, Collagen degradation, Pulmonary Fibrosis Idiopathic Signaling Pathway, Wound Healing Signaling Pathway, GP6 Signaling Pathway, and Hepatic Fibrosis/ Hepatic Stellate Cell Activation pathways. Chronic alcohol is known to impact the expression of collagen in the amygdala and across the body due to alcohol-related fibrosis [141]. This further strengthens the claim that binge-like drinking can worsen negative outcomes in alcohol-associated diseases.

### Wnt signaling and molecular alcohol tolerance

Because Wnt-signaling is necessary for rapid molecular alcohol tolerance [20,21,25] we looked at potentially dysregulated genes associated to this pathway (Fig 6,7). While we found few genes suggesting this connection we did identify some key changes in the synaptogenesis signaling pathways which had several dysregulated cadherin genes (CDH1, CDH17, CDH19, CDH22) these genes have all been associated to Wnt signaling and E-Cadherin in particular is well known prevent internalization of β-Catenin suggesting alteration in Wnt signaling persist long past their role in early alcohol molecular tolerance [150–152]. It also possessed a reduction of SNY3 which could suggest an increase in BK activity [153]. The Calcium signaling pathway was also notable as changes in intracellular calcium could affect BK channel signaling. Here we found 2 dysregulated SERCA pumps (ATP2A1, ATP2B3) which could impact ER calcium however they are regulated in opposite directions making it difficult to assess the nature of the impact [154]. We also found dysregulated transient receptor potential cation channels (Trpc2, TRPC5, TRPM8) but they differed in their directionality [155]. Other genes include a group of potential BK channels regulators (SNA12, TNFSF13, and NOX1) suggesting an alteration in BK signaling [156–158], ITPR1 can also increase β-catenin signaling which could allude to a connection with molecular alcohol tolerance [159], and AANAT which is linked to BK activity [160].

### Fear and stressors

Interestingly The opioid signaling pathway revealed a small group of genes associated with fear and anxiety (ADCY9, ITPR1) which could alter fear response and be responsible for the previously observed extinction deficits [161,162]. CHRM5, a gene thought to control the duration of dopamine release from the VTA was also found to be dysregulated which suggests a possible desensitization to dopamine takes place [163]. Perhaps the most important gene from the opioid pathway was OPRM1 which is known to modulate the hedonic effects of alcohol in humans [164], and alter how Pavlovian stimuli affect dopamine signaling [165].

### Translational relevance

Overall, our findings indicate that binge-like alcohol exposure impairs extinction learning, alters early drinking behaviors, and disrupts multiple neuronal pathways, independently of the mechanisms underlying early molecular alcohol tolerance. These results could lead to the identification of key molecular players, and previously unidentified molecular pathways relevant to the study of AUD. Transcriptomics can be easily translated to human models and represents an important bridge between pre and post clinical research [166]. In fact, post-mortem studies in humans with AUD have shown promising overlap with preclinical transcriptomics [167]. Similar is the case for stress-related disorders [168]. As such this could be relevant for guiding potential clinical research. This study allows for a deeper insight into changes in mRNA expression profiles as a result of both binge-like alcohol exposure and it effects on extinction learning. While we are currently focusing on male C57BL/6 mice due to the clear effects of alcohol exposure we will be evaluating the changes in female mice in future studies [22].

## Supporting information

S1 Fig Differential expression analysis EtOH vs. Control of e-cadherin associated genes. Diagram representing differentially expressed E-Cadherin associated genes and their known/predicted interactions sorted by cellular location. Figures produced from QIAGEN IPA software – open-access CC-BY 4.0 license for purposes of publication.
(TIF)

S1 Table Examples of differentially expressed EtOH vs. Control of e-cadherin associated genes. (TIF)

S2 Fig Differential expression analysis EtOH vs. Control of opioid signaling associated genes. Diagram representing differentially expressed opioid signaling associated genes and their known/predicted interactions sorted by cellular location. Figures produced from QIAGEN IPA software – open-access CC-BY 4.0 license for purposes of publication.
(TIF)

S2 Table Examples of differentially expressed EtOH vs. Control of opioid signaling genes. (TIF)

S3 Fig Differential expression analysis EtOH vs. Control of calcium signaling associated genes. Diagram representing differentially expressed calcium signaling associated genes and their known/predicted interactions sorted by cellular location. Figures produced from QIAGEN IPA software – open-access CC-BY 4.0 license for purposes of publication.
(TIF)

S3 Table Examples of differentially expressed EtOH vs. Control of calcium signaling genes. (TIF)

S4 Fig Differential expression analysis EtOH vs. Control of collagen biosynthesis associated genes. Diagram representing differentially expressed collagen biosynthesis associated genes and their known/predicted interactions sorted by cellular location. Figures produced from QIAGEN IPA software – open-access CC-BY 4.0 license for purposes of publication.
(TIF)

S4 Table Examples of differentially expressed EtOH vs. Control of ethanol exposure. (TIF)

S5 Fig Differential expression analysis EtOH vs. Control of GP6 associated genes. Diagram representing differentially expressed GP6 associated genes and their known/predicted interactions sorted by cellular location. Figures produced from QIAGEN IPA software – open-access CC-BY 4.0 license for purposes of publication.
(TIF)

S5 Table Examples of differentially expressed EtOH vs. Control of GP6 signaling genes. (TIF)

S6 Fig Differential expression analysis EtOH vs. Control of CREB signaling associated genes. Diagram representing differentially expressed CREB signaling associated genes and their known/predicted interactions sorted by cellular location. Figures produced from QIAGEN IPA software – open-access CC-BY 4.0 license for purposes of publication.
(TIF)

S6 Table Examples of differentially expressed EtOH vs. Control of CREB signaling genes. (TIF)

S7 Fig Differential expression analysis EtOH vs. Control of GPCR signaling associated genes. Diagram representing differentially expressed GPCR signaling associated genes and their known/predicted interactions sorted by cellular location. Figures produced from QIAGEN IPA software – open-access CC-BY 4.0 license for purposes of publication.
(TIF)

S7 Table Examples of differentially expressed EtOH vs. Control of GPCR signaling genes. (TIF)

S8 Fig Differential expression analysis EtOH vs. Control of synaptogenesis associated genes. Diagram representing differentially expressed synaptogenesis associated genes and their known/predicted interactions sorted by cellular location. Figures produced from QIAGEN IPA software – open-access CC-BY 4.0 license for purposes of publication.
(TIF)

S8 Table Examples of differentially expressed EtOH vs. Control of synaptogenesis associated genes. (TIF)

S9 Fig Differential expression analysis EtOH vs. Control of dopamine signaling associated genes. Diagram representing differentially expressed dopamine signaling associated genes and their known/predicted interactions sorted by cellular location. Figures produced from QIAGEN IPA software – open-access CC-BY 4.0 license for purposes of publication.
(TIF)

S9 Table Examples of differentially expressed EtOH vs. Control of dopamine signaling genes. (TIF)
