# Supplementary figures and images for: Comparative mRNA profile analysis from NAc of adolescent male mice after binge-like alcohol exposure eliciting deficits in context fear extinction learning
Source: PLoS One. 2025 Jun 25;20(6):e0322576. doi: 10.1371/journal.pone.0322576 (PMC12193044; doi:10.1371/journal.pone.0322576)

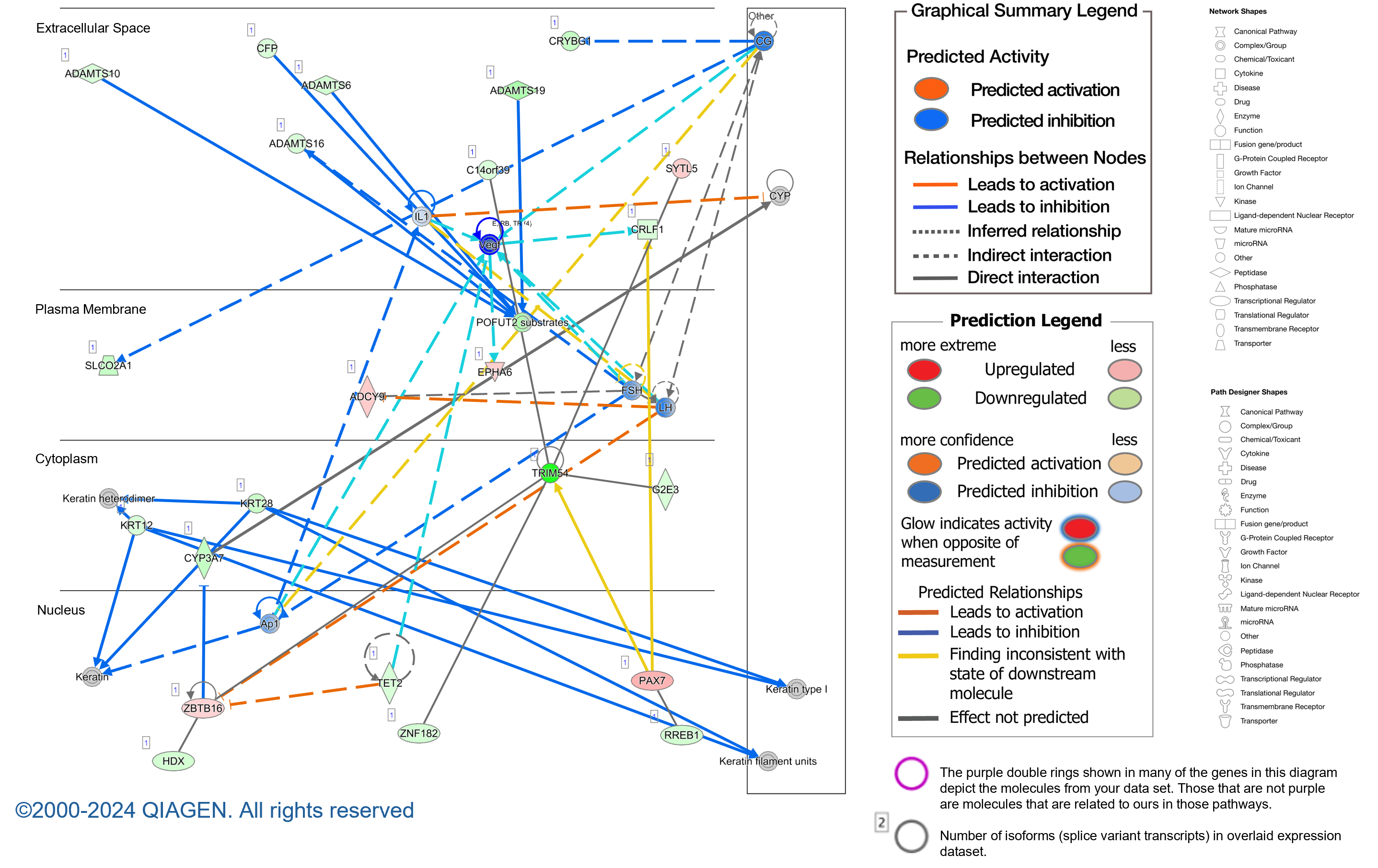

Supplement: S1 Fig — Diagram representing differentially expressed E-Cadherin associated genes and their known/predicted interactions sorted by cellular location. Figures produced from QIAGEN IPA software – open-access CC-BY 4.0 license for purposes of publication. (TIF) [file pone.0322576.s001.tif]

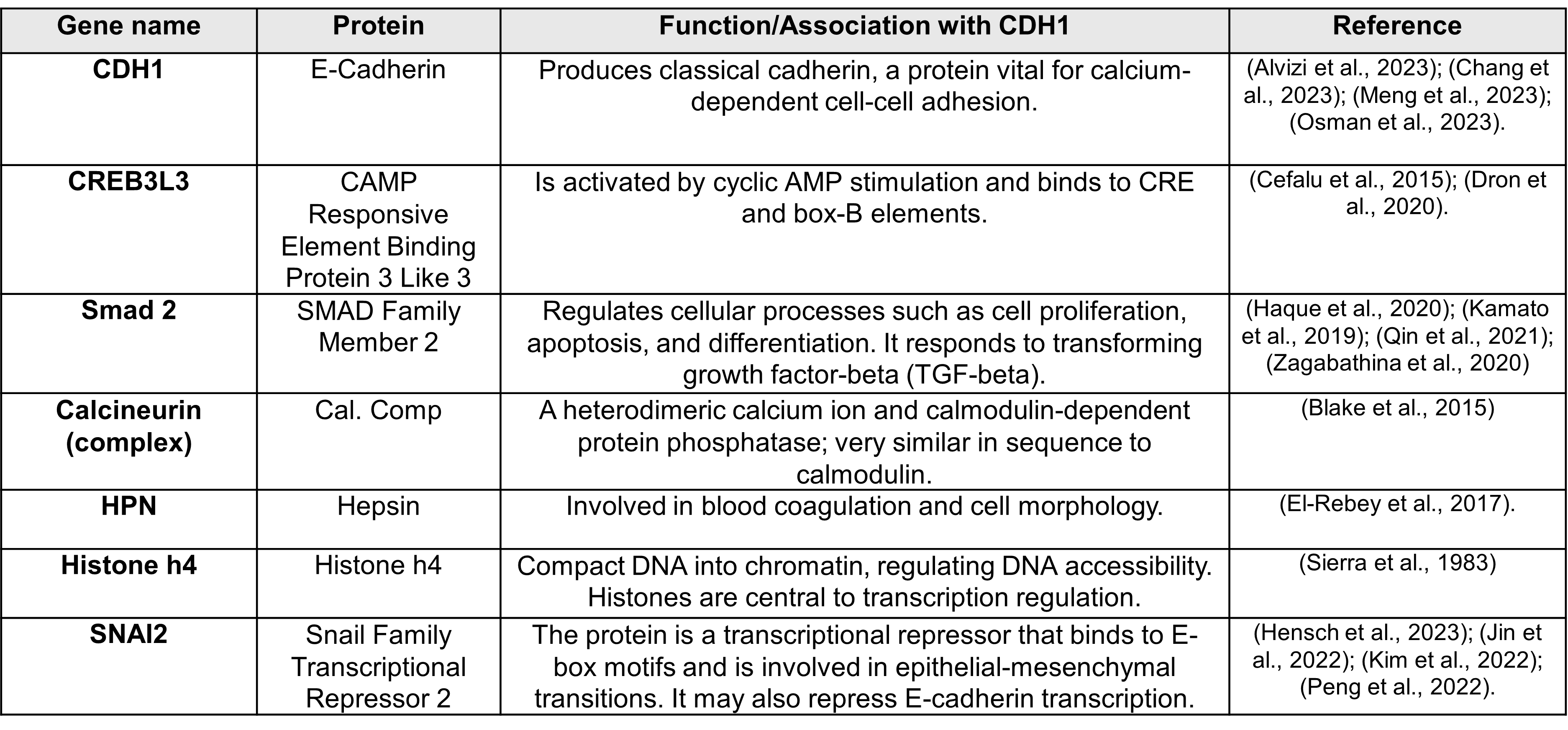

Supplement: S1 Table — (TIF) [file pone.0322576.s002.tif]

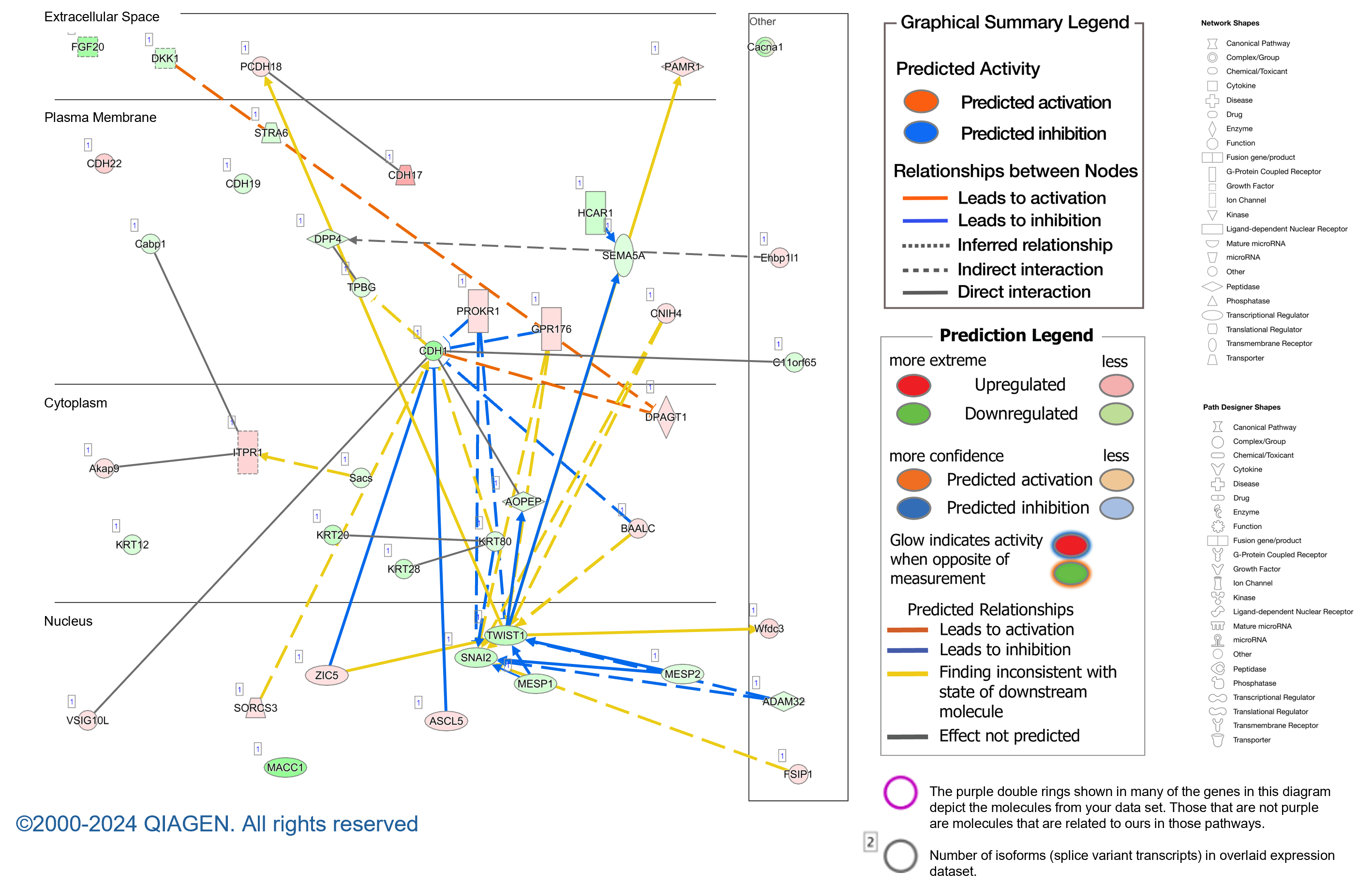

Supplement: S2 Fig — Diagram representing differentially expressed opioid signaling associated genes and their known/predicted interactions sorted by cellular location. Figures produced from QIAGEN IPA software – open-access CC-BY 4.0 license for purposes of publication. (TIF) [file pone.0322576.s003.tif]

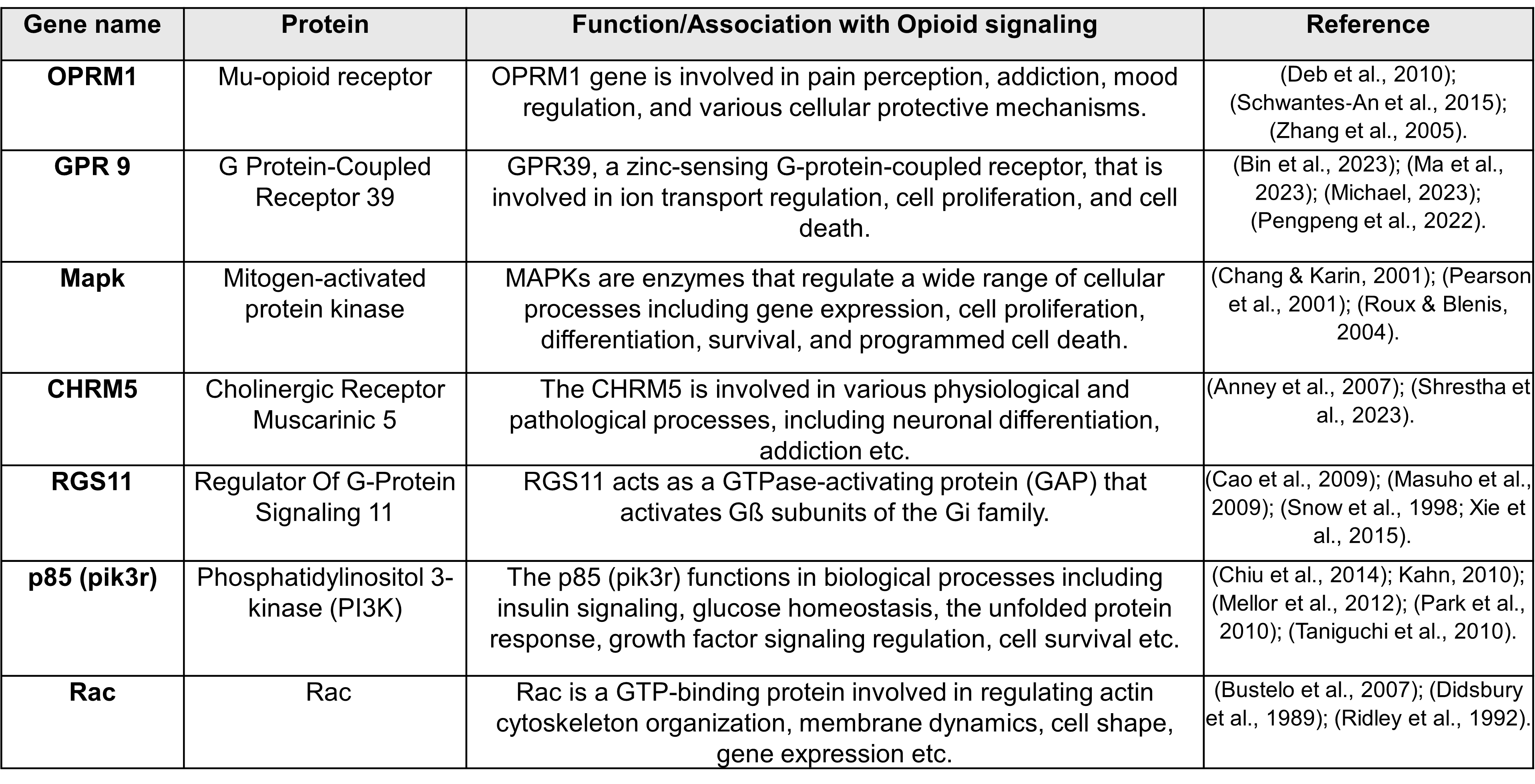

Supplement: S2 Table — (TIF) [file pone.0322576.s004.tif]

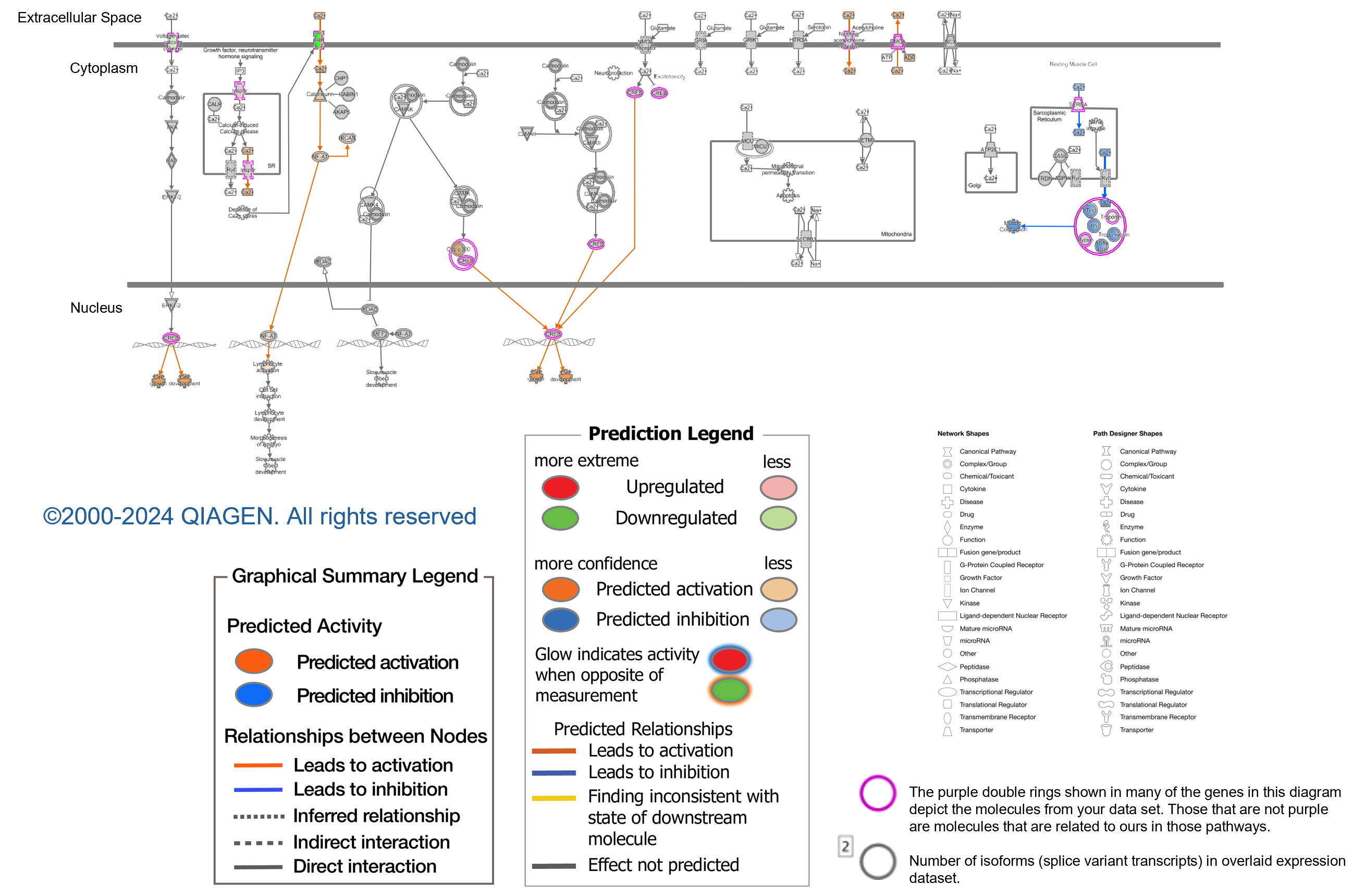

Supplement: S3 Fig — Diagram representing differentially expressed calcium signaling associated genes and their known/predicted interactions sorted by cellular location. Figures produced from QIAGEN IPA software – open-access CC-BY 4.0 license for purposes of publication. (TIF) [file pone.0322576.s005.tif]

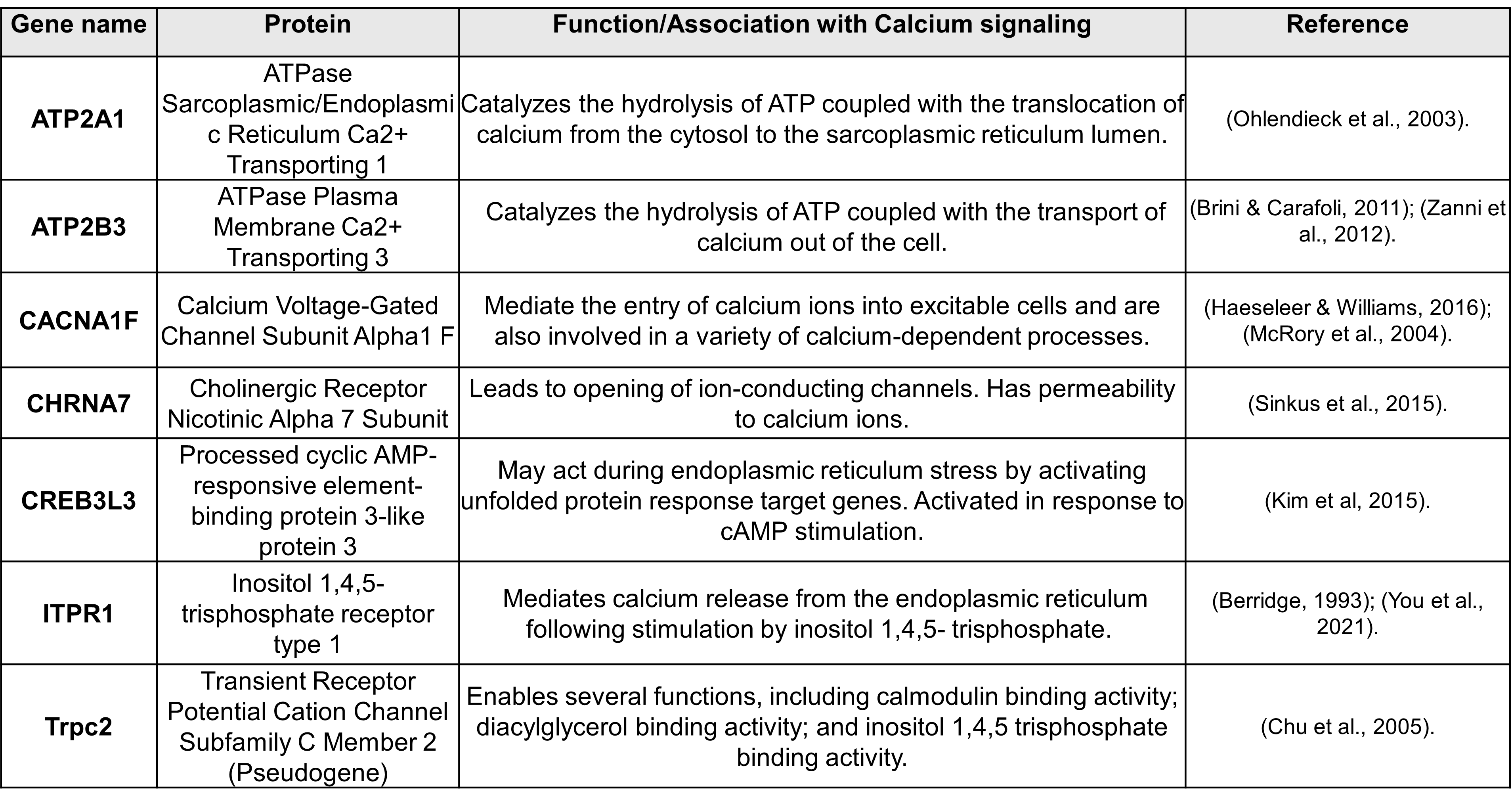

Supplement: S3 Table — (TIF) [file pone.0322576.s006.tif]

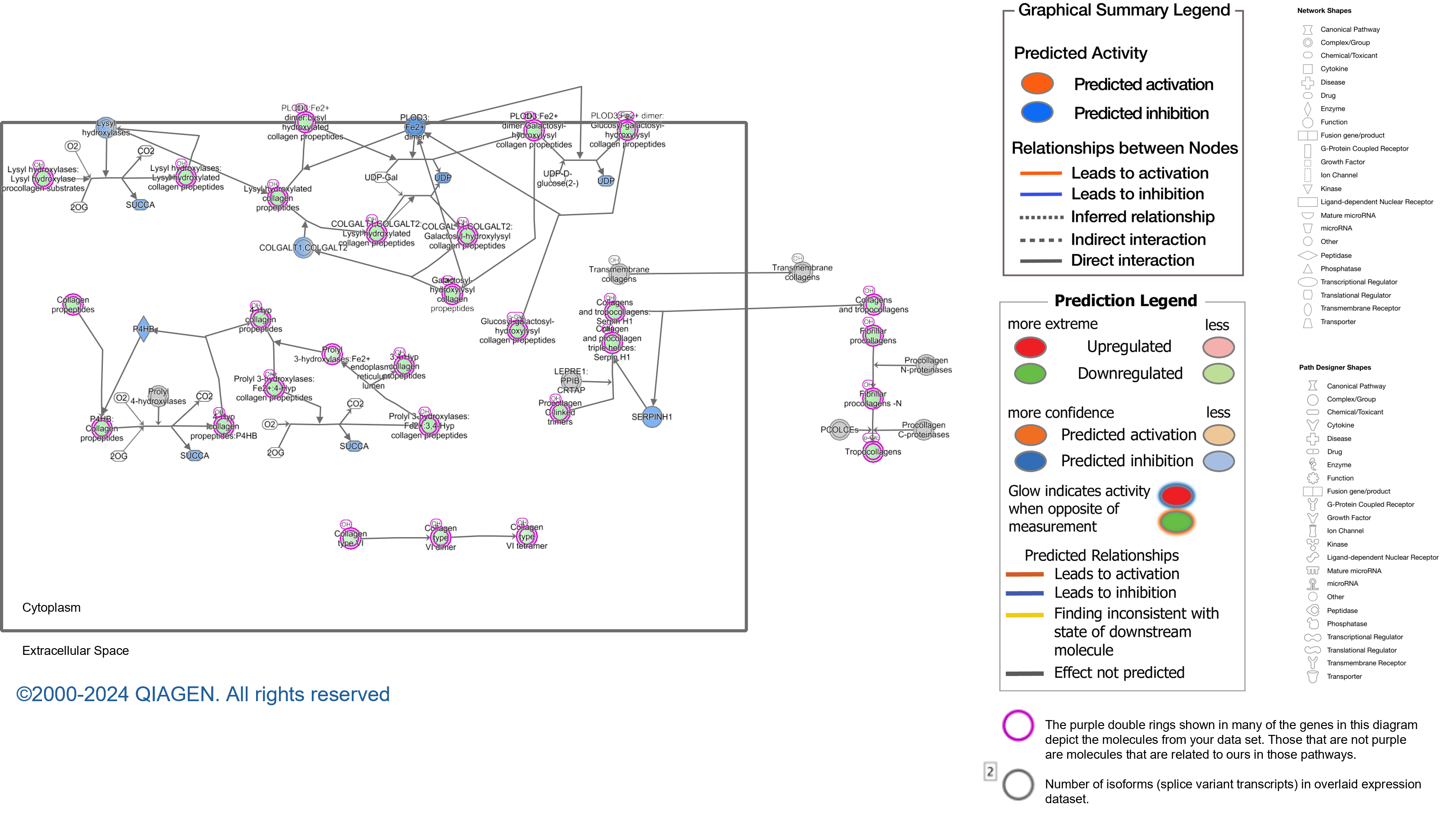

Supplement: S4 Fig — Diagram representing differentially expressed collagen biosynthesis associated genes and their known/predicted interactions sorted by cellular location. Figures produced from QIAGEN IPA software – open-access CC-BY 4.0 license for purposes of publication. (TIF) [file pone.0322576.s007.tif]

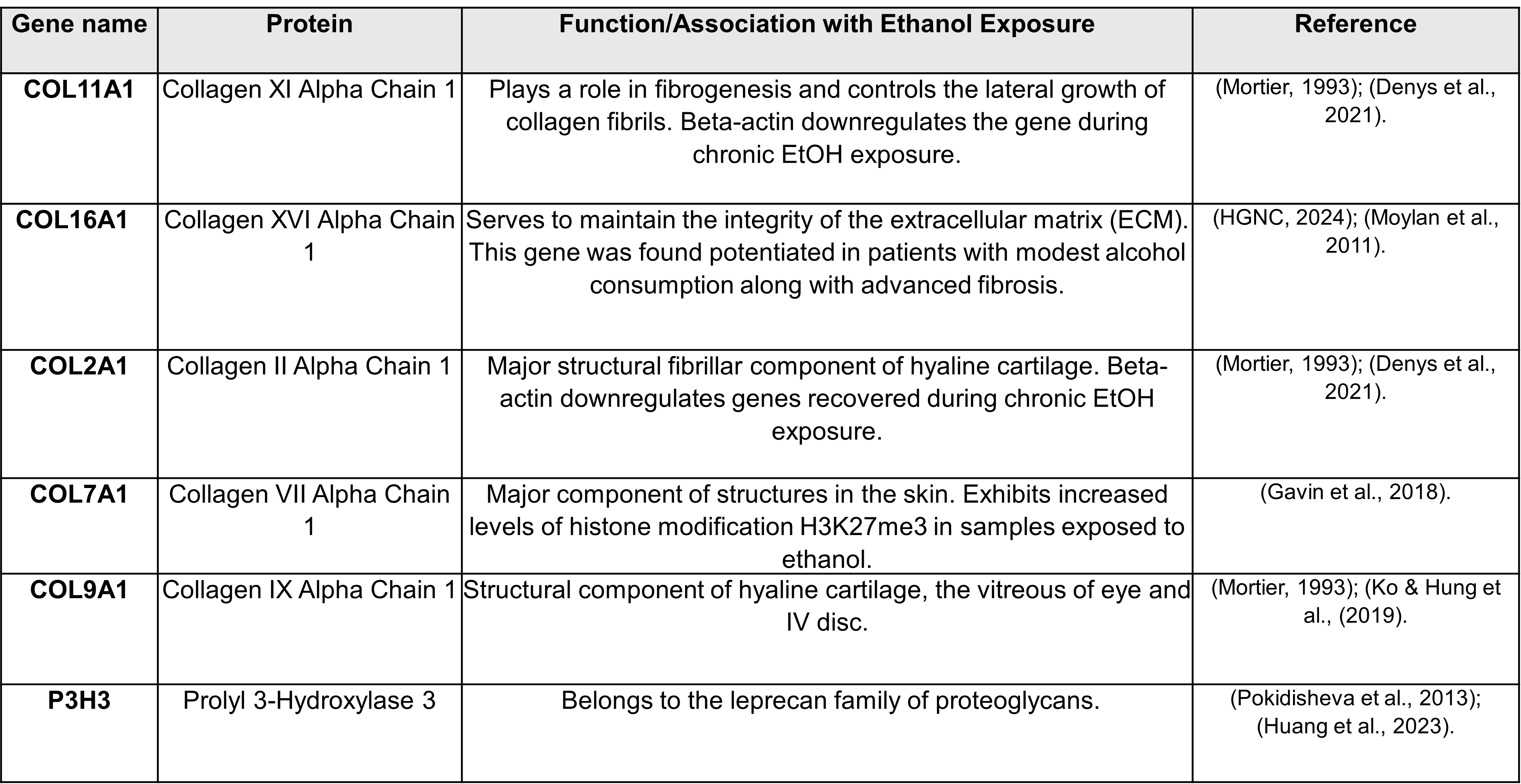

Supplement: S4 Table — (TIF) [file pone.0322576.s008.tif]

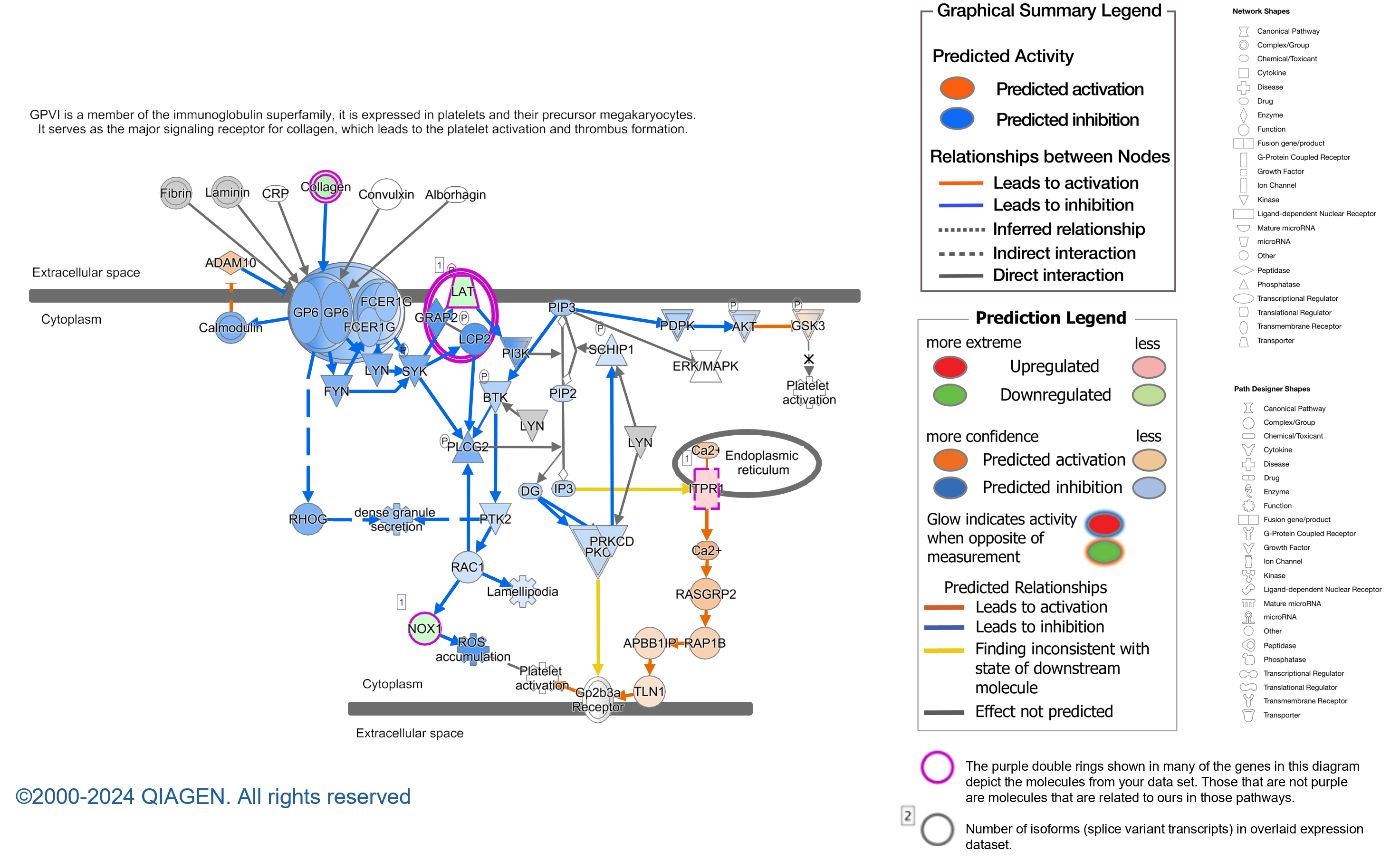

Supplement: S5 Fig — Diagram representing differentially expressed GP6 associated genes and their known/predicted interactions sorted by cellular location. Figures produced from QIAGEN IPA software – open-access CC-BY 4.0 license for purposes of publication. (TIF) [file pone.0322576.s009.tif]

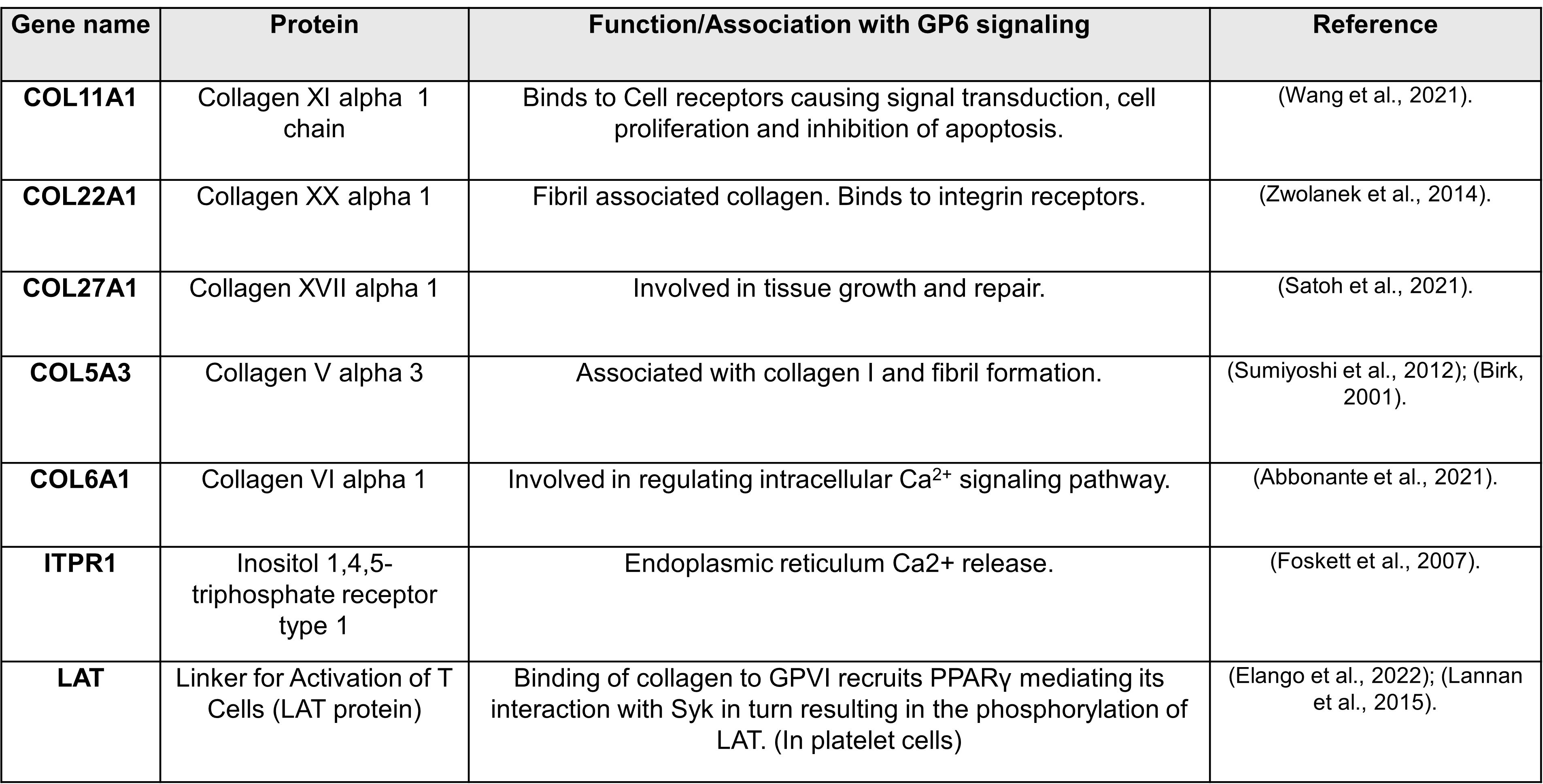

Supplement: S5 Table — (TIF) [file pone.0322576.s010.tif]

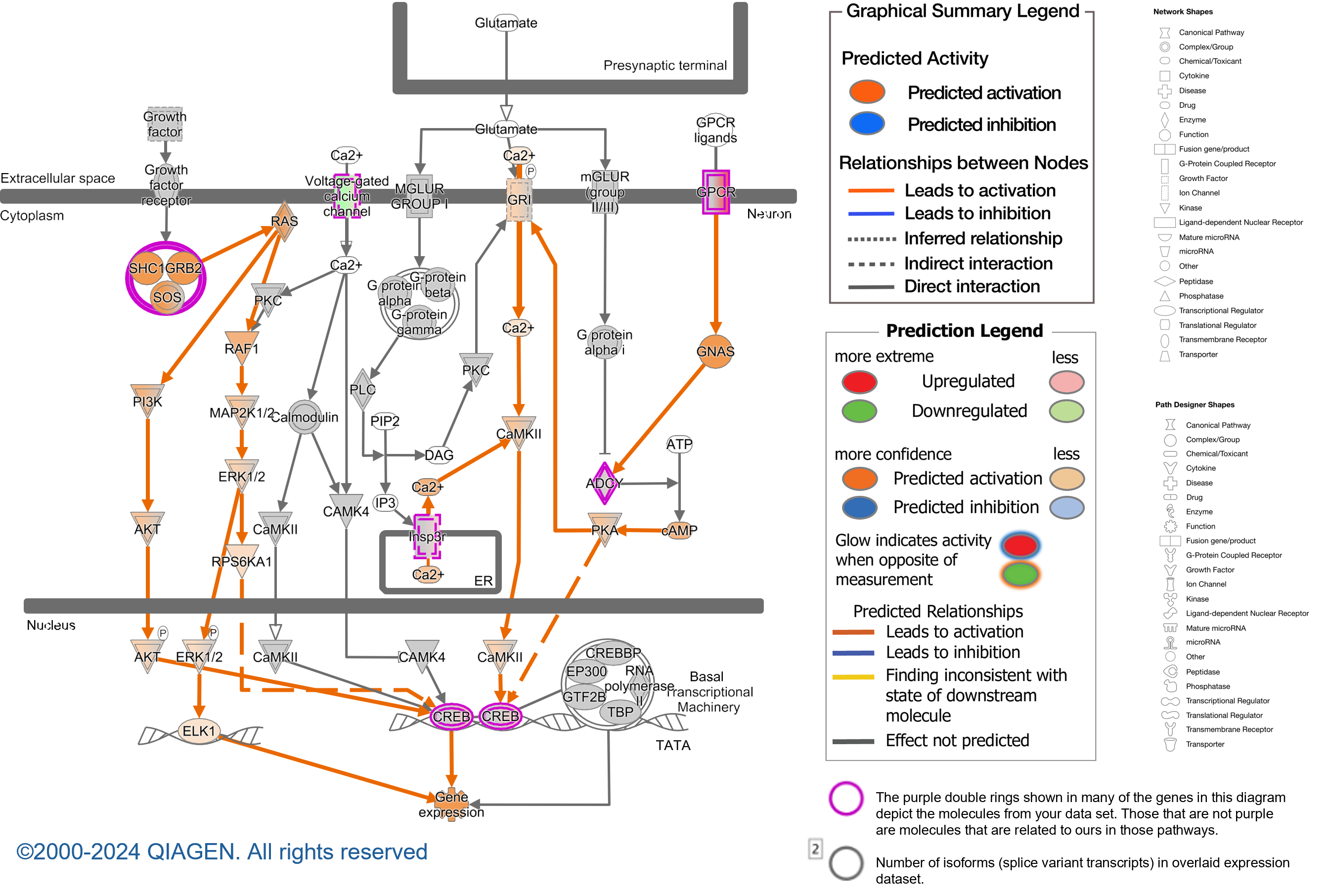

Supplement: S6 Fig — Diagram representing differentially expressed CREB signaling associated genes and their known/predicted interactions sorted by cellular location. Figures produced from QIAGEN IPA software – open-access CC-BY 4.0 license for purposes of publication. (TIF) [file pone.0322576.s011.tif]

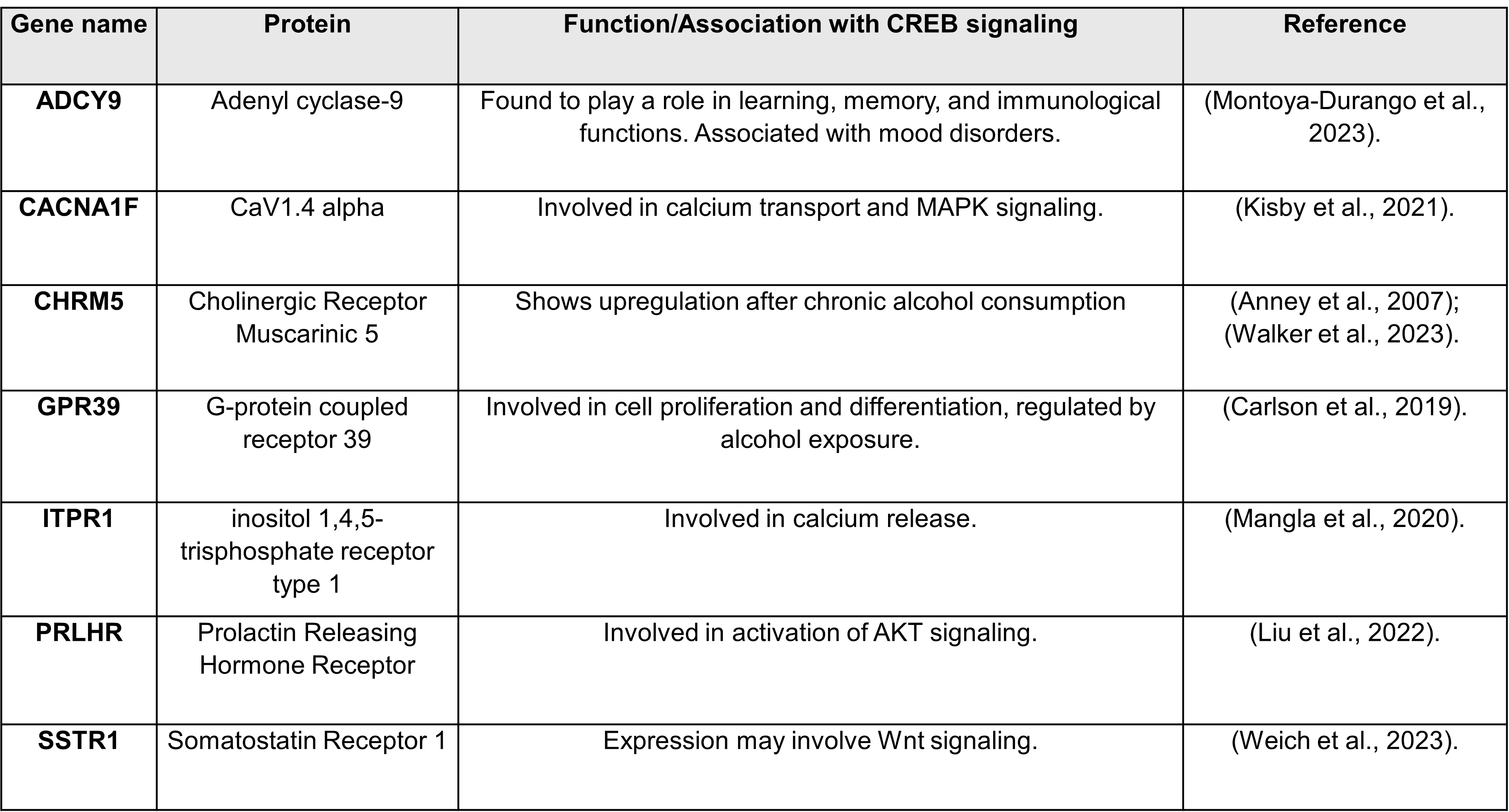

Supplement: S6 Table — (TIF) [file pone.0322576.s012.tif]

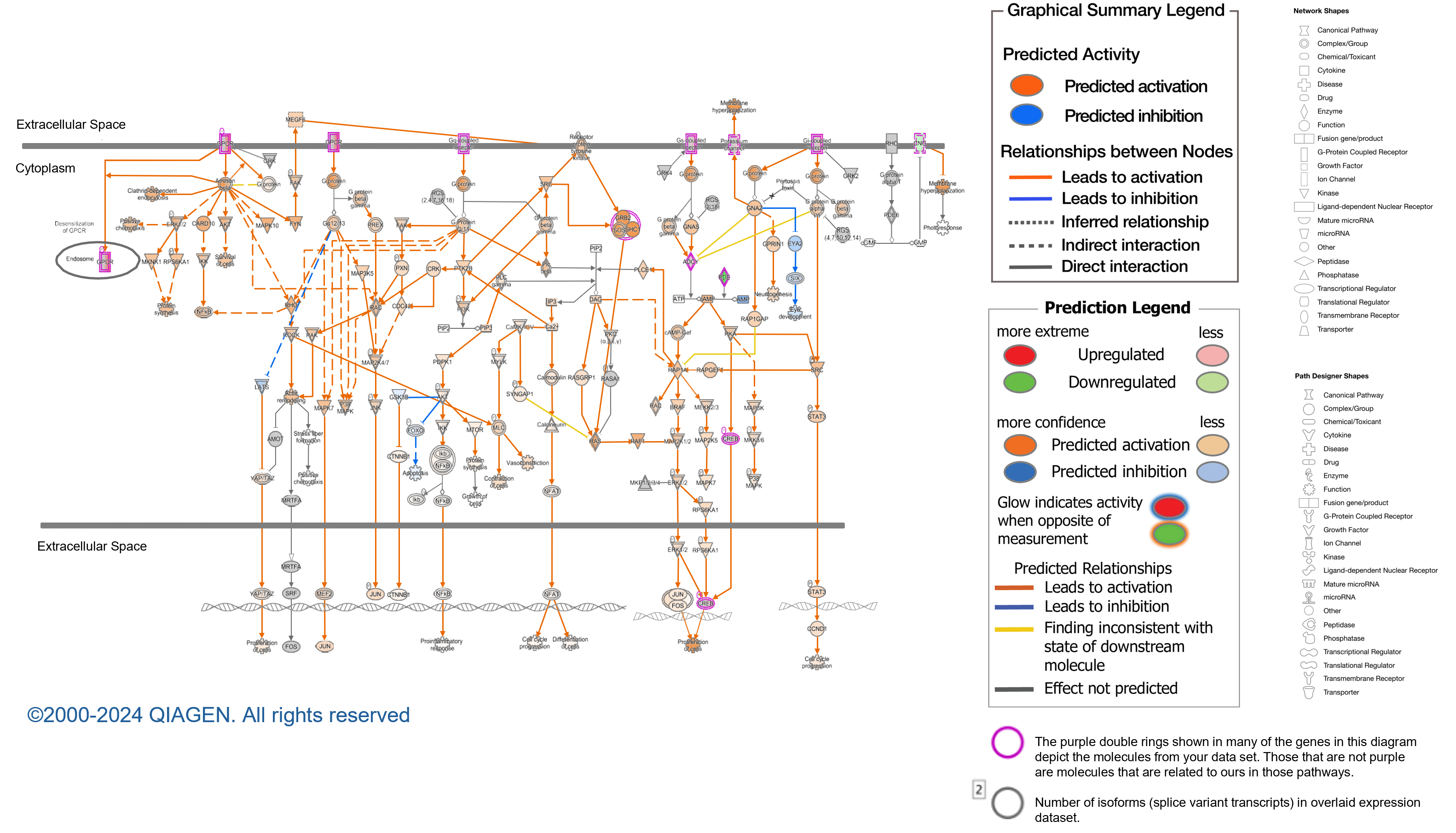

Supplement: S7 Fig — Diagram representing differentially expressed GPCR signaling associated genes and their known/predicted interactions sorted by cellular location. Figures produced from QIAGEN IPA software – open-access CC-BY 4.0 license for purposes of publication. (TIF) [file pone.0322576.s013.tif]

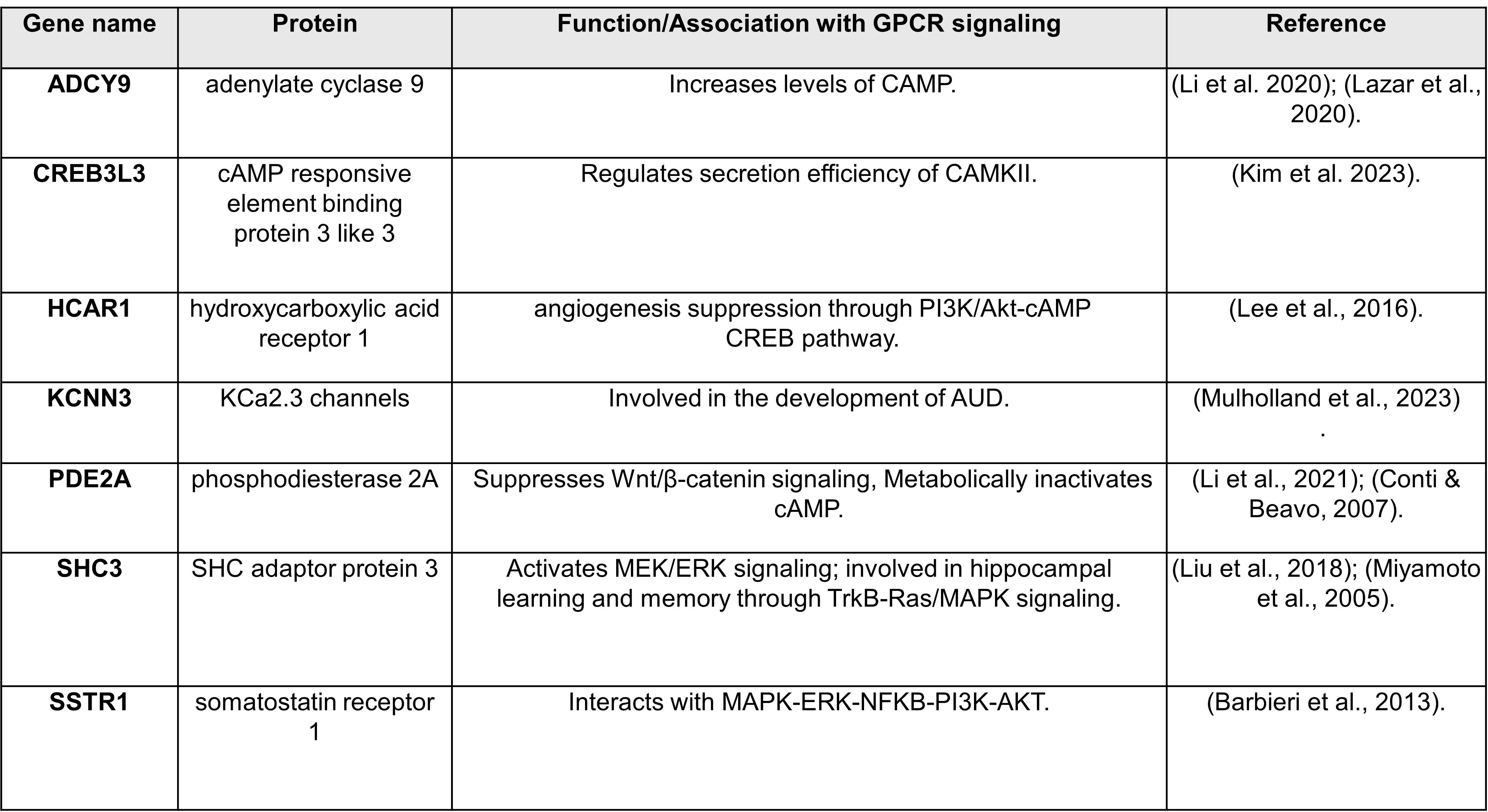

Supplement: S7 Table — (TIF) [file pone.0322576.s014.tif]

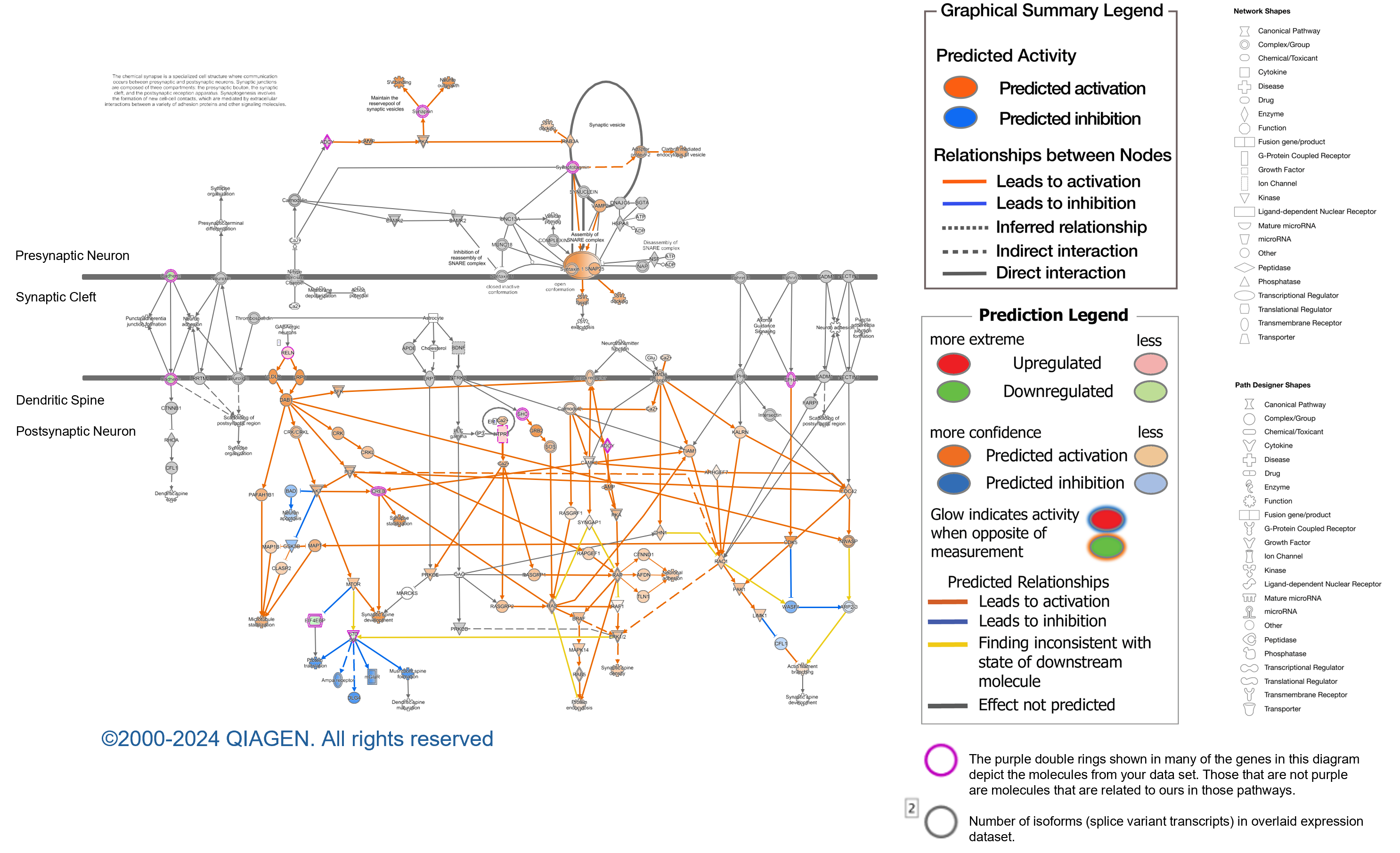

Supplement: S8 Fig — Diagram representing differentially expressed synaptogenesis associated genes and their known/predicted interactions sorted by cellular location. Figures produced from QIAGEN IPA software – open-access CC-BY 4.0 license for purposes of publication. (TIF) [file pone.0322576.s015.tif]

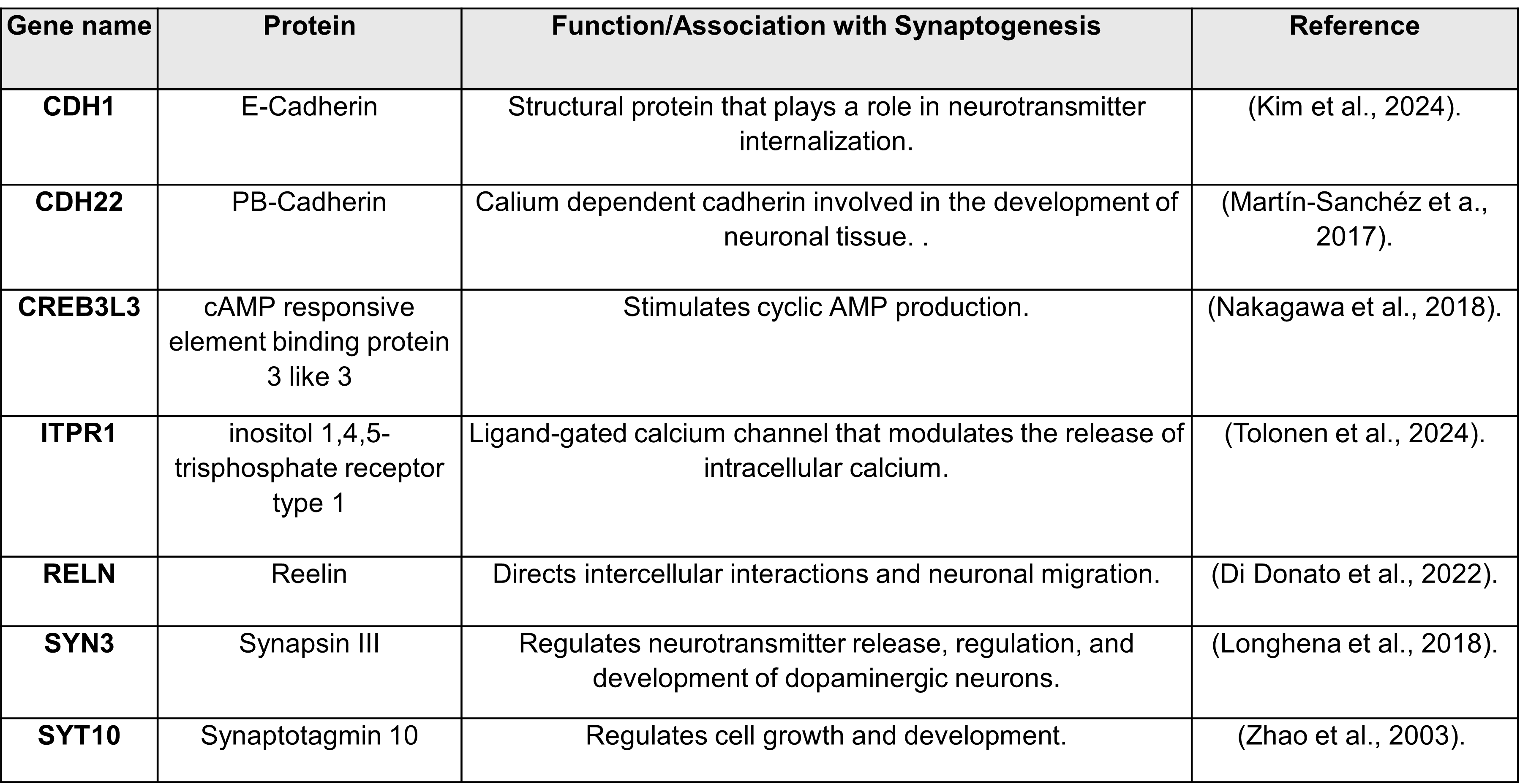

Supplement: S8 Table — (TIF) [file pone.0322576.s016.tif]

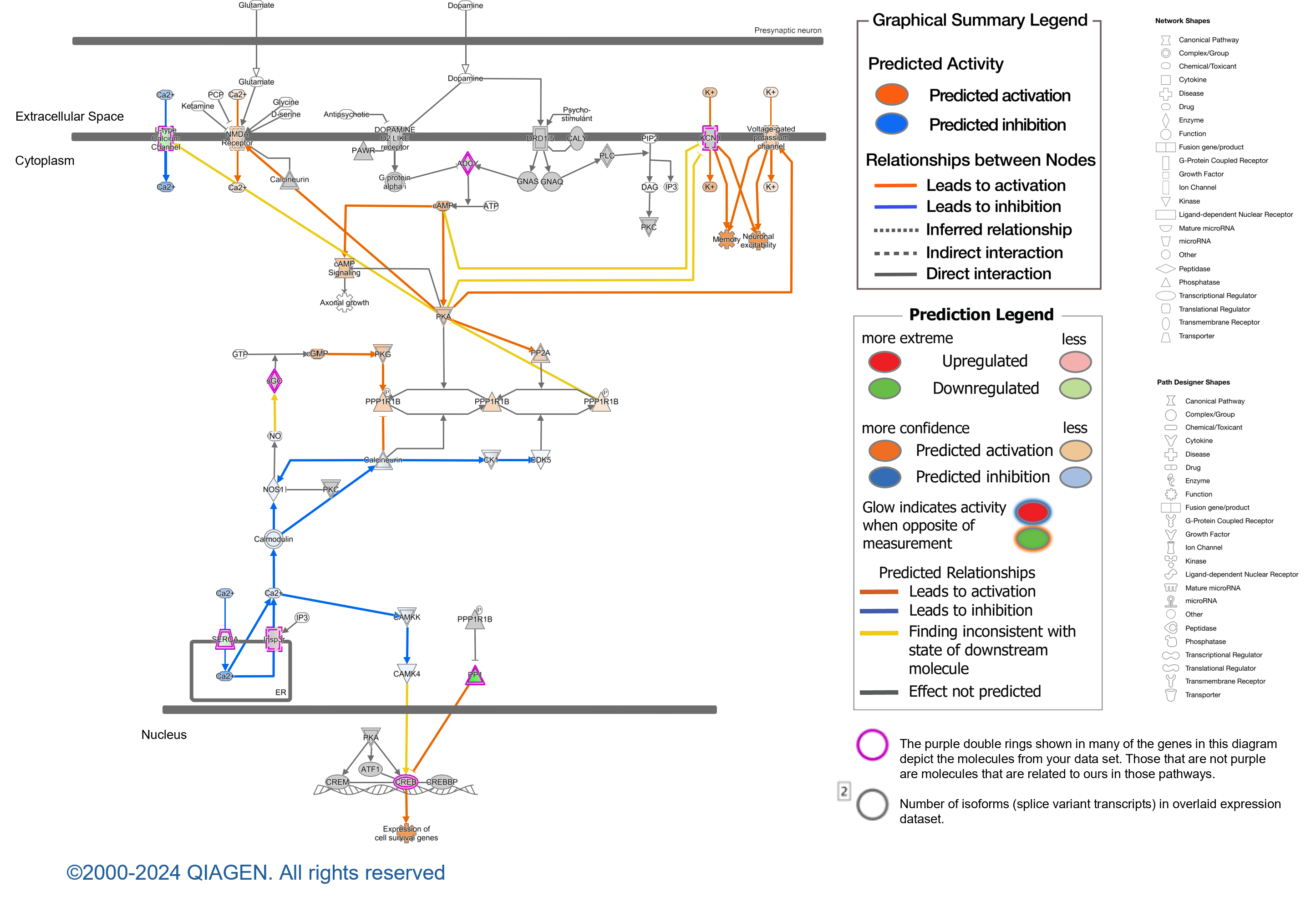

Supplement: S9 Fig — Diagram representing differentially expressed dopamine signaling associated genes and their known/predicted interactions sorted by cellular location. Figures produced from QIAGEN IPA software – open-access CC-BY 4.0 license for purposes of publication. (TIF) [file pone.0322576.s017.tif]

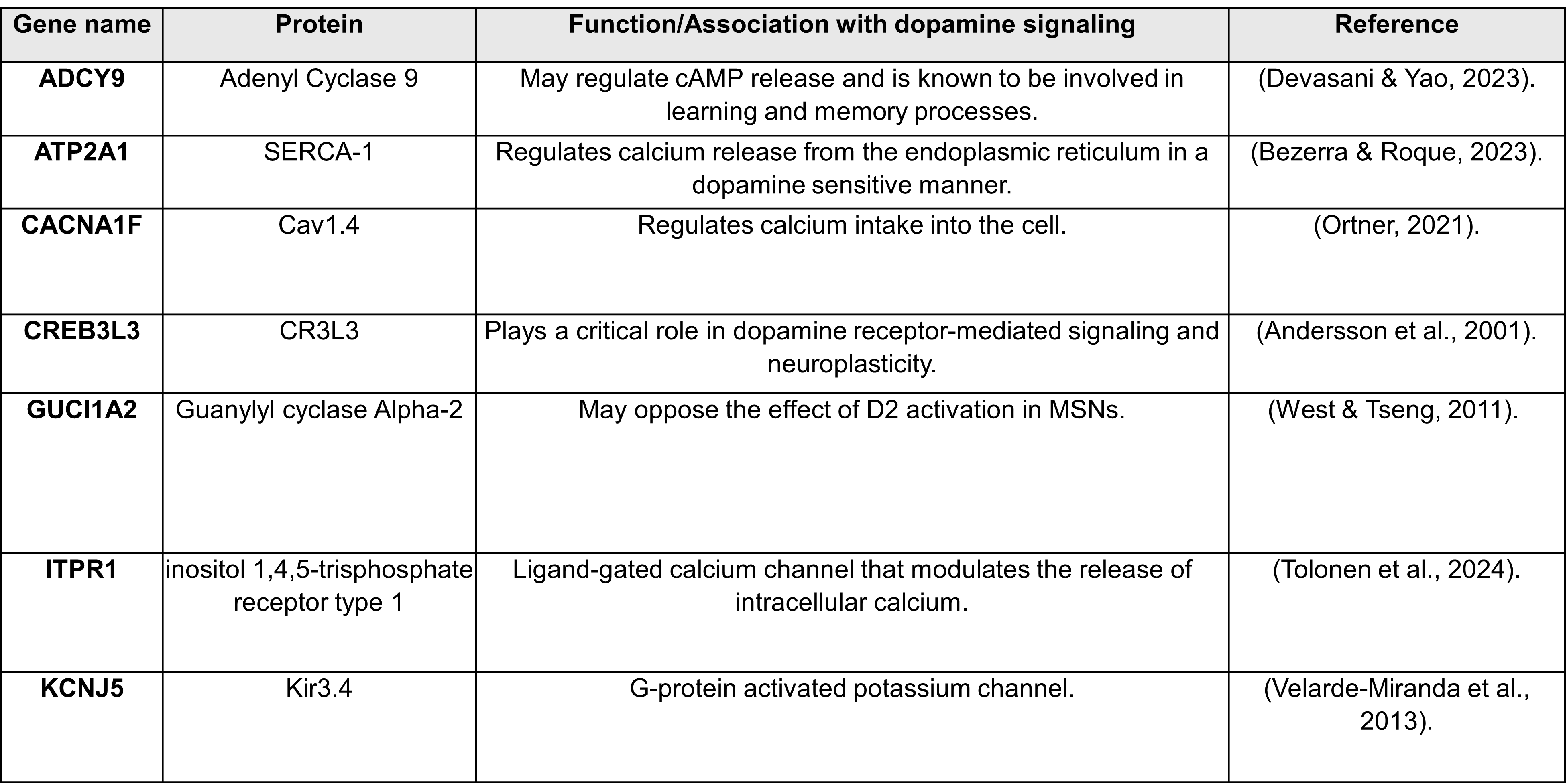

Supplement: S9 Table — (TIF) [file pone.0322576.s018.tif]
